# The physiological foundation of extinction improvement by tDCS over the ventromedial prefrontal cortex (vmPFC) in healthy humans: an fMRI study

**DOI:** 10.1038/s41398-026-04190-4

**Published:** 2026-06-26

**Authors:** Harleen Chhabra, Yuanbo Ma, Erhan Genç, Michael A. Nitsche, Fatemeh Yavari

**Affiliations:** 1 Department of Psychology and Neurosciences, Leibniz Research Centre for Working Environment and Human Factors, Dortmund, Germany; 2 Department of Psychology, Ruhr University Bochum, Bochum, Germany; 3 Bielefeld University, University Hospital OWL, Protestant Hospital of Bethel Foundation, University Clinic of Psychiatry and Psychotherapy, Bielefeld, Germany; 4 German Center for Mental Health (DZPG), Bochum, Germany

**Keywords:** Neuroscience, Learning and memory

## Abstract

A growing body of research highlights the fear extinction model as a key framework for understanding the pathology of anxiety disorders and developing therapeutic interventions. Functional imaging studies link successful extinction recall and reduced amygdala reactivity with ventromedial prefrontal cortex (vmPFC) activity, while dysfunctional vmPFC activity is associated with anxiety pathology. Enhancing vmPFC activity through transcranial direct current stimulation (tDCS) has thus emerged as a promising approach to augment cognitive behavioral therapy (CBT) and therapeutic exposure therapies for anxiety disorders. In this study, anodal tDCS was applied over the vmPFC during the extinction learning phase of a 3-day fear acquisition/extinction paradigm (anodal tDCS: 21, sham tDCS: 23 participants). Skin conductance response (SCR) and Blood Oxygenation Level Dependent (BOLD) brain activity were recorded simultaneously. Healthy participants who received real tDCS showed significantly improved extinction recall compared to the sham tDCS group, as indicated by the SCR. Fear acquisition activated core regions of the fear network, including the anterior cingulate cortex, amygdala, orbitofrontal cortex, and insular cortex. During extinction learning, real tDCS activated both fear and safety networks (particularly the posterior cingulate and middle temporal cortex) during early extinction, while the sham tDCS group showed activity only in the fear network. Furthermore, a decoupling of the vmPFC with fear network regions with real tDCS during extinction was observed, indicating strengthened inhibitory control. These findings suggest that anodal tDCS over the vmPFC promotes early activation of the safety network, enhancing extinction learning and consolidation. This suggests its translational potential as an adjunctive intervention for anxiety disorders.

## Introduction

Anxiety disorders are among the most prevalent mental health conditions worldwide, with a 55% rise in affected individuals since 1990 [1]. Core features include excessive fear, anxiety, avoidance of threats, functional impairment, and treatment challenges [2]. Despite current evidence-based interventions, such as cognitive-behavioral therapy (CBT) and serotonergic medications, 30–60% of individuals retain symptoms, and relapse rates reach 50% among young adults within six years [3, 4].

A growing body of research identifies the fear extinction model as a cornerstone for understanding anxiety disorder pathology and for developing therapeutic mechanisms [5]. Grounded in Pavlovian conditioning, fear extinction reduces conditioned responses when a conditioned stimulus (CS) is repeatedly presented without the unconditioned stimulus (US) [6]. As the core mechanism of exposure therapy, extinction is widely used in anxiety and anxiety-related disorders, such as PTSD (post-traumatic stress disorder), and OCD (obsessive-compulsive disorder), which share overlapping activation in key fear circuitry regions [7].

Skin conductance response (SCR) is a common and reliable physiological index of fear extinction in humans, reflecting sympathetic arousal [8]. Furthermore, magnetic resonance imaging (MRI) during fear extinction studies examines brain activation, connectivity, and dynamics during fear learning and extinction. Neural responses involve fear and safety networks: the fear network shows higher activation by conditioned than neutral stimuli, while the safety network shows the reverse pattern. Fear network regions include the anterior cingulate cortex (ACC), insular cortex, amygdala, cerebellum, and the supplementary motor area [9–14]. Coactivation of these regions has been reported in fear-learning studies [10–12]. On the other hand, proposed safety network regions include the angular gyrus, ventromedial prefrontal cortex (vmPFC), hippocampus, orbitofrontal cortex (OFC), posterior cingulate cortex (PCC), parahippocampus, insular cortex, middle temporal gyrus, cerebellum, caudate, and primary somatosensory cortex [11, 15].

The ventromedial prefrontal cortex (vmPFC) plays a pivotal role in fear extinction and is central to the top-down regulation of the amygdala, one of the crucial brain regions responsible for the expression and modulation of fear [6, 15]. Dysfunctions in vmPFC-amygdala connectivity are involved in the persistence and severity of anxiety disorders [16]. Additionally, reduced vmPFC activation during extinction recall has been observed in individuals with PTSD, often accompanied by increased activity in other fear-related areas such as the dorsal anterior cingulate cortex (dACC) [17]. Furthermore, previous studies observed that the vmPFC plays a critical role in emotion regulation [18, 19]. Reduced activity of the bilateral vmPFC region [17, 20] and reduced connectivity of the right vmPFC and amygdala [21] have been linked to anxiety disorders. Animal studies have reported that while lesions in the infralimbic cortex (IL, homologous to the human vmPFC) impaired the retention of extinction [22] and contextual processing [23], stimulation of the IL during extinction enhanced learning and retention of extinction [24–26]. Therefore, enhancing vmPFC activity in humans could also be a promising strategy to augment CBT/exposure therapies for anxiety disorders.

Transcranial direct current stimulation (tDCS) modulates cortical excitability using low-intensity electrical currents, with anodal and cathodal stimulation generally linked to increased and decreased excitability, respectively. Short-term effects result from transient shifts in resting membrane potentials, altering neuronal firing [27]. Longer stimulation produces lasting after-effects, partly mediated by GABAergic and NMDA receptor mechanisms resembling characteristics of long-term potentiation (LTP) and depression (LTD) [28, 29]. LTP has been proposed as one of the physiological mechanisms underlying learning [30] and tDCS has been reported to facilitate learning and memory formation [31, 32], potentially involving synaptic mechanisms.

Similar to the above-mentioned IL stimulation animal studies, targeting the vmPFC with tDCS in humans has shown promise in modulating fear extinction. Enhanced fear extinction and recall are observed when anodal tDCS is applied during extinction learning over the vmPFC [33–36]. Although a recent meta-analysis did not reveal a significant effect of anodal tDCS on extinction, the analysis was limited by the small number of studies and heterogeneous study designs in terms of task design and tDCS stimulation parameters [26]. Targeting the medial or ventromedial PFC however, enhances fear extinction [34, 35], whereas targeting the dorsal PFC inhibits fear memory consolidation [37]. Moreover, stimulation duration and intensity significantly influence both immediate and lasting effects [38, 39].

tDCS parameters have been previously reported to significantly impact the immediate and aftereffects of interventions [38, 39]. Extinction studies to date have used different intervention protocols with respect to current intensities, stimulation duration, and electrode placement and size, resulting in heterogeneous results. Therefore, in the current study, we chose intervention parameters to address these limitations while being able to draw parallels with the previous literature. Unlike previous studies, we stimulated the vmPFC bilaterally, as both hemispheres play critical roles in emotion regulation and anxiety disorders [17–21, 40]. We used a focused tDCS approach to increase the focality of the stimulation to the vmPFC and reduce diffusion of the electric field to other regions involved in fear and extinction learning [41, 42]. We stimulated for the complete duration of the extinction phase (10 min), unlike other studies, which continued stimulation after the extinction trials ended, possibly resulting in over-generalization of fear to non-reinforced stimuli [36], stimulated selectively after extinction, possibly resulting in inhibited extinction recall [43], or stimulated either selectively during the first or second block of extinction, resulting in incomplete extinction [33]. Lastly, we selected 2 mA tDCS intensity as it aligns with protocols used in treatment-focused studies tackling anxiety and related disorders, facilitating mechanistic comparisons.

The present study evaluated the role of anodal tDCS over the vmPFC in modulating extinction learning and recall. We assessed physiological responses with SCR and extinction-related neural changes in the context of fear and safety networks. To capture temporal dynamics, SCR and MRI analyses were divided into early and late blocks [44, 45]. We hypothesized that vmPFC stimulation induces LTP-like plasticity [46, 47], enhancing extinction learning and recall as shown by reduced SCRs, particularly in early blocks. We further predicted increased vmPFC activation, similar to prior motor cortex stimulation studies that reported increased activation with anodal stimulation [48, 49]. Furthermore, we predicted that for extinction and recall, the real tDCS group would show reduced activity of fear-related and increased activity of safety-related regions, and there would be stronger vmPFC decoupling with fear-related regions [24–26, 50]. Given that functional decoupling has been linked to reduced neural responses [51] increased decoupling under real tDCS would reflect an inhibitory effect of vmPFC activation on the fear network, thereby reducing fear responses.

## Methodology

### Participants

Sixty-eight (33 females) right-handed, non-smoking, and healthy adults aged between 18–40 years were recruited via online and offline advertisements. All were native or proficient (≥B1) German speakers and naïve to the fear and extinction task. Handedness was confirmed with the Edinburgh Handedness Inventory [52], and their health status was confirmed by an in-house medical doctor. Exclusion criteria included current or any history of neurological or psychiatric disorders, epilepsy, metabolic disorders, having metal implants anywhere in the body, brain or skull injury, aphasia, color-blindness, claustrophobia, pregnancy or lactation, or alcohol, nicotine (smoking) or drug dependency. Before the experiment, the participants completed the Depression Anxiety Stress Scale 21 (DASS-21) [53], Anxiety Sensitivity Index 3 (ASI-3) [54], and the Brief Experimental Avoidance Questionnaire (BEAQ) [55]. Higher scores indicated greater expression of the respective traits. The protocol was approved by the ethics committee of the Leibniz Research Centre for Working Environment and Human Factors, was in accordance with the Declaration of Helsinki, and all participants provided informed consent and received financial compensation.

Data from 24 participants were excluded: Of the participants, 16 had a mean CS- response > mean CS+ response during the acquisition phase. The mean was calculated for each participant by averaging the SCR values across all CS+ trials and CS– trials during the acquisition phase [56]. This exclusion criterion is similar to that used by other studies [57, 58], where non-learners were defined as participants who showed either a negative discrimination (<0 µS) or no discrimination (=0 µS) of the SCRs in the acquisition phase. The exclusion of 23% (n = 16) of the participants based on the above-mentioned criteria aligns with proportions reported in other studies, where exclusion rates have been shown to range from 2% to 74% [57]. An additional 8 participants had noisy data. The data were considered noisy if the mean SCR value was <0.02µS [58–60] because at this low level, it was difficult to distinguish random fluctuations from the real SCR.

The fear extinction task employed in this study is a well-established paradigm for investigating fear learning and extinction and has been used in our previous work [61–63]. An a priori power analysis was conducted using G*Power 3.1, with SCR as the primary outcome measure. For a repeated-measures ANOVA with a within-between interaction design (tDCS as the between-subject factor; CS type [CS+, CS-], and Block [early, late] as within-subject factors), assuming an alpha level of 0.05 and power of 0.95, a total sample size of 36 participants was required to detect a medium effect size (f = 0.25). The final sample of 44 participants, therefore, exceeded this requirement and was considered adequately powered.

### Fear conditioning and extinction task

**Fig. 1:**
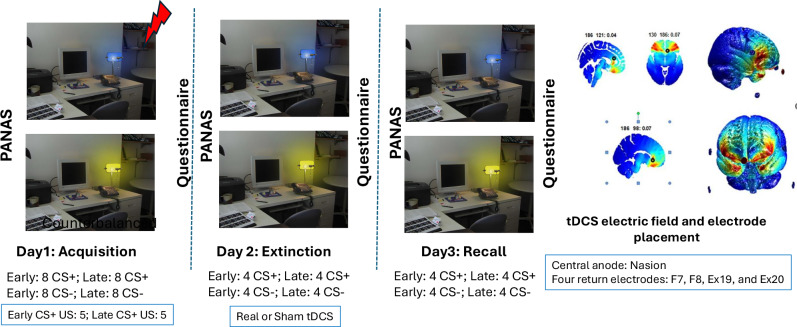
Differential AAA fear conditioning/extinction paradigm with the event blocks and tDCS electrode placement. Conditioned stimuli (CS) were presented in the same context, a picture of a desk (“office”), and were indexed by either a blue (CS+) or yellow (CS-) light lamp. The unconditioned stimulus (US) was an electric stimulation applied to the right index and middle fingers. Fear acquisition, extinction learning, and extinction recall were performed on three consecutive days. The task trials were divided into the early and late blocks. Acquisition had 16 CS and 5 CS + US in early and late blocks, respectively. Both extinction and recall had 8 CS and no US in early and late blocks, respectively. During extinction, participants received either real or sham tDCS over the vmPFC. Focal stimulation of the vmPFC (via 2 cm diameter round electrodes) was computationally modeled to maximize the electric field (EF) strength in the right and left vmPFC regions, while minimizing EF at other regions of the fear network (bilateral DLPFC, anterior cingulate cortex and cerebellum), and the modelling results show the targeted engagement of the vmPFC predicted from the modelled e-fields. The Positive and Negative Affect Schedule (PANAS) questionnaire was administered each day before starting the experiment to assess the emotional state before the task. The tDCS questionnaire was administered after each session to assess the online tDCS side-effects and tDCS blinding, and additionally before extinction and recall sessions to assess tDCS side-effects that appeared within 24 h after tDCS administration. Also, participants rated valence, emotional arousal, fear, and US expectancy for CS+ and CS- trials after the task on each day.

The three phases of the Pavlovian fear conditioning paradigm, namely fear acquisition, extinction, and extinction recall [6] were administered over three consecutive days (approximately 24 h apart). A three-day design was used because extinction immediately after acquisition may promote fear unlearning rather than forming extinction memory [64]. The task design was identical to that of Milad et al. [6] and Ma et al. [62] (Fig. 1) with a consistent CS across all phases (AAA) and MRI-adjusted trial timings. Each trial began with 1.2 s of neutral context, followed by 12 s CS + /CS- presentation. In reinforced trials, the US was delivered 11.9 s after CS+ onset and co-terminating with the CS + . Intertrial intervals (white cross on grey background) were jittered between 19.2–22.8 s duration. Acquisition included 16 CS+ (10 reinforced, 62.5%) and 16 CS− trials. Extinction and recall each included 8 CS+ and 8 CS- trials with no US (see Supplementary document section 1.1 for details).

#### tDCS

tDCS targeting the vmPFC was delivered with a Starstim stimulator (NeuroElectrics, Barcelona, Spain). Computational modeling using ROAST (https://www.parralab.org/roast/) [2] determined the optimal montage (see Supplementary Document section 1.2 for details). The anode was placed over the nasion, and four return electrodes (cathodes) at F7, F8, Ex19, and Ex20 (Fig. 1). A reference electrode was placed over the right mastoid to reduce resistance and stabilize stimulation inside the MRI. To enhance blinding, topical anesthetic cream (EMLA®, 2.5% lidocaine, 2.5% prilocaine) was applied to all electrode sites [65].

During the 10-min extinction phase, 2 mA tDCS was applied with 30-s ramp-up and 30-s ramp-down at the start and end of the intervention. Sham stimulation ramped up to 2 mA for 30 s before ramping down immediately afterwards, and the device was then disconnected. Circular 2 cm diameter electrodes were used, yielding a current density of 0.637 mA/cm² at the anode and 0.159 mA/cm² at the return electrodes. This protocol ensures blinding without inducing lasting neurophysiological effects [66, 67].

Participants were blinded to their stimulation group and unaware of the task phase associated with stimulation. tDCS electrodes were placed on the scalp for the three task phases to maintain blinding (for scores of tDCS blinding refer to Supplementary document Tables S4a, S4b, and S4c).

#### Skin conductance response (SCR) acquisition

SCR during the experiment was measured using a Brain Vision Brain Amp ExG and the Brain Vision Recorder (Brain Products GmbH, Gliching, Germany). Two SCR electrodes were placed over the middle phalanx of the right index and middle finger. EDALYT gel (Easy Cap GmbH, Wörthsee, Germany) was applied to the electrodes. The data were acquired with a sampling frequency of 1000 Hz, a range of 3.277 mV, a high cutoff filter of 250 Hz, and a resolution of 152.6 uV.

#### Questionnaires

Participants were required to answer three questionnaires in immediate connection with the experimental protocol. The details of the administered questionnaires are explained in the supplementary document section 1.3.

#### MRI acquisition

All images were acquired using a 3 T scanner at IfADo (MAGNETOM 3 T, Siemens Healthcare GmbH, Erlangen, Germany) using a 64-channel head coil. The scanner headspace was appropriately cushioned (below the head and on the sides) to avoid head movement during scanning.

##### Anatomical imaging

For coregistration of functional data, T1-weighted high-resolution anatomical images were acquired (TR = 2530 ms, TE = 2.36 ms, flip angle = 7°, 176 slices, matrix size = 256 mm × 256 mm, resolution = 1 mm × 1 mm × 1 mm). The scanning duration was around 6 min.

##### Task-based imaging

Whole-brain BOLD activity was acquired during the fear conditioning task image presentation using a multiband (MB) sequence provided by the Center for Magnetic Resonance Research (CMRR), University of Minnesota, Minneapolis. It was based on accelerated T2*-weighted echo planar imaging (TR = 1200 ms, TE = 31.40 ms, MB factor = 6, flip angle = 52°, 78 slices, matrix size = 211 mm × 211 mm, partial Fourier factor = 6/8, EPI factor = 132, number of slices = 78, resolution = 1.6 mm × 1.6 mm × 1.6 mm). The scanning duration of each phase of the fear conditioning and extinction task was the same as the task duration (acquisition = 20 min and extinction and recall = 10 min).

### Procedure

At the beginning of each phase, the participants were prepared with electrodes (tDCS, SCR, and aversive stimuli) and given task instructions. They were informed that any CS-US pattern they noticed would remain unchanged [62]. After completing the PANAS, they entered the scanner and were instructed to stay still and focused. On the acquisition day (Day 1), US intensity was determined before task MRI: it started low and was increased until it was perceived as “highly uncomfortable but not painful” (real tDCS: 48.84 ± 15.82 V; sham: 44.01 ± 22.46 V). The US was then applied at this level. After the task, participants rated valence, arousal, fear, and US expectancy, and completed the tDCS adverse effects questionnaire. For extinction, recall days, and follow-up (email/phone), participants also reported any post-tDCS adverse effects.

### SCR analysis

SCR data were down-sampled from 1000 Hz to 100 Hz using Brain Vision Analyzer and exported to MATLAB (R2021b, The MathWorks, Natick, MA, USA). A semi-automated peak detection algorithm was used [68], defining SCR amplitude as the maximum trough-to-peak change within 1–12.5 s post-CS onset, with a minimum amplitude of 0.02 μS and rise time of 0.5 s [56]. To account for inter-individual variability of SCR responses, a mean-correction procedure was employed [69]. Specifically, for each participant, the mean SCR across all trials was calculated and subsequently subtracted from every raw SCR value.

SCRs were analyzed in early and late blocks: acquisition (16 CS+ and 16 CS-) was split into trials 1–8 (early) and 9–16 (late); extinction and recall (8 CS+ and 8 CS-) were split into trials 1–4 (early) and 5–8 (late).

### Task-based imaging data analysis

The functional images were distortion-corrected using ANTs (https://stnava.github.io/ANTs/). These corrected images were analysed in the FSL toolbox FEAT (http://www.fmrib.ox.ac.uk/fsl, version 6.0.1). Preprocessing included motion correction, 50 Hz high-pass filtering, spatial smoothing (6 mm FWHM), and registration to T1 and MNI space. First-level GLMs generated activation maps (CS+ > CS−, CS− > CS+) for acquisition, extinction, and recall, with events modeled using a gamma HRF. Analyses were split into early (first half) and late (second half) blocks. Second-level analyses included one-sample t-tests (acquisition) and independent-samples t-tests (real vs sham tDCS during extinction and recall), with random-effects estimation using randomise with 5000 permutations, and TFCE (p < 0.05). Results were visualized in FSLeyes, with anatomical labels from the Harvard-Oxford atlas and Cerebellar Atlas in MNI152 space [70]. Region of interest (ROI) analysis of the vmPFC (Harvard-Oxford 2006/2007 frontal medial mask as provided in FSL/FSLeyes) was performed to test real > sham tDCS effects during extinction and recall. The vmPFC was approximated with the Harvard–Oxford Frontal Medial Cortex region (max-probability atlas, 75% threshold, 2 mm MNI152 space; Suplementary document Fig. S3). At this stringent threshold, the mask was largely restricted to the ventral medial prefrontal and medial orbitofrontal cortex, consistent with the canonical vmPFC core. The resulting region also encompassed the vmPFC cluster observed in functional activation analyses and the tDCS stimulation target. Lastly, psychophysiological interaction (PPI) analyses with vmPFC as a seed were done to examine connectivity during extinction and recall, testing the real > sham tDCS effects.

### Statistical analysis

All analyses were conducted in IBM SPSS Statistics 26 (IBM Corp., Armonk, NY, USA). Chi-square tests and independent-samples t-tests assessed demographics and psychological measures (DASS-21, ASI-3, BEAQ). SCR data were analyzed with mixed-model ANOVAs, with Group (real, sham) as a between-subject and Block (early, late) and Stimulus (CS+, CS−) as within-subject factors. This approach is widely adopted in fear extinction research [34, 71] and allows each phase’s learning dynamics to be assessed in detail. Greenhouse–Geisser corrections were applied in case of sphericity violations identified by the Mauchly test, and significant effects in the ANOVAs were followed by Fisher’s LSD post hoc tests (*p* < 0.05).

## Results

### Demographics

At baseline, the two tDCS groups did not significantly differ, as indicated by the respective independent samples t-tests and chi-square tests (Supplementary document Table S1).

### Absolute SCRs elicited by CS+ and CS−

#### Fear acquisition phase (Day 1)

**Fig. 2:**
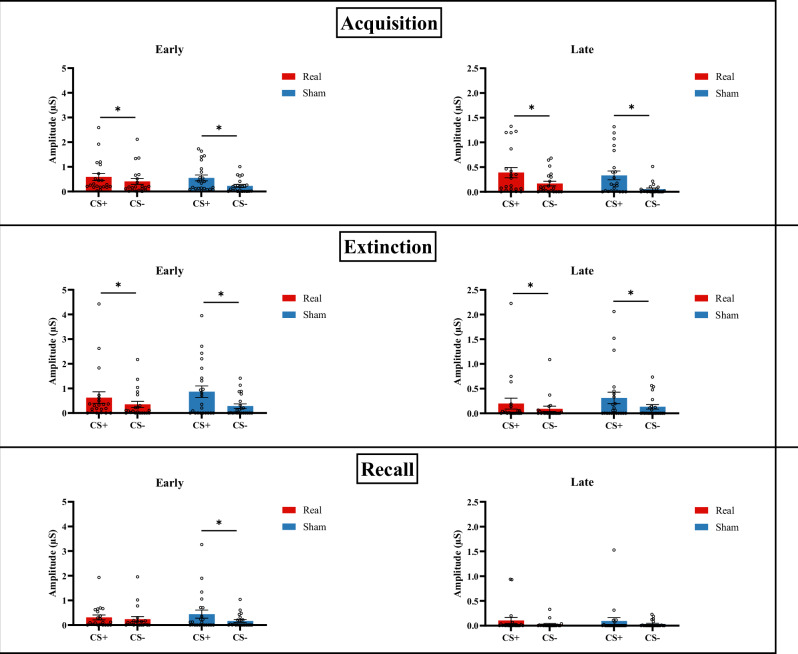
The SCRs in response to the CS+ and CS- for each block across acquisition, extinction, and recall phases are shown. Bar colors represent different groups, and error bars indicate the standard error of the mean. During the acquisition phase, there was a significant main effect of stimulus, and the post hoc test revealed that both tDCS groups showed significantly larger SCR in response to the CS+ than the CS- in the early and late blocks. During the extinction phase, stimulus × block a significant interaction was present, both tDCS groups showed a significantly larger SCR in response to the CS+ than the CS- and this effect was greater in the early compared to the late block. During the recall phase, there was a significant group × stimulus × block interaction, and the sham group had a significantly larger SCR in response to the CS+ than the CS- in the early block. Asterisks (* p < 0.05) mark significant results of post-hoc comparisons.

The mixed model ANOVA revealed a significant main effect of Stimulus (F (1, 42) = 35.834, p < 0.001). Post hoc tests indicated that the CS+ elicited significantly larger SCRs compared to the CS- (p < 0.001). Furthermore, the main effect of Block was significant (F (1, 42) = 15.469, p < 0.001). Post hoc comparisons showed that block 1 produced significantly larger SCRs compared to block 2 (p < 0.001). No other significant main effects or interactions were found (Table 1, Fig. 2 and Supplementary document Table S2, Fig. S1).

**Table 1:** Results of the mixed-model ANOVAs comparing SCRs across the acquisition, extinction, and recall phases.

	Factors	F value	df, error	p	η_p_^2^
**Acquisition**	Group	0.73	1, 42	0.39	0.02
	Stimulus	35.83	1, 42	**<0.001**	0.46
	Block	15.47	1, 42	**<0.001**	0.27
	Stimulus × Group	1.35	1, 42	0.25	0.03
	Block × Group	0.05	1, 42	0.82	0.00
	Stimulus × Block	0.00	1, 42	0.97	0.00
	Stimulus × Block × Group	0.55	1, 42	0.46	0.01
**Extinction**	Group	0.26	1, 42	0.61	0.01
	Stimulus	14.03	1, 42	**0.001**	0.25
	Block	19.59	1, 42	**<0.001**	0.32
	Stimulus × Group	1.54	1, 42	0.22	0.04
	Block × Group	0.00	1, 42	0.96	0.00
	Stimulus × Block	7.78	1, 42	**0.008**	0.16
	Stimulus × Block × Group	1.27	1, 42	0.27	0.03
**Recall**	Group	0.01	1, 42	0.89	0.00
	Stimulus	7.06	1, 42	**0.01**	0.14
	Block	17.72	1, 42	**<0.001**	0.29
	Stimulus × Group	1.106	1, 42	0.30	0.026
	Block × Group	0.081	1, 42	0.78	0.002
	Stimulus × Block	3.727	1, 42	0.06	0.081
	Stimulus × Block × Group	4.374	1, 42	**0.04**	0.094

*df*, degrees of freedom.

#### Extinction learning phase (Day 2)

The mixed model ANOVA revealed significant main effects of Stimulus (F (1, 42) = 14.029, p = 0.001) and Block (F (1, 42) = 19.595, p < 0.001). Additionally, the Stimulus × Block interaction was significant (F (1, 42) = 7.783, *p* = 0.008). Post-hoc tests revealed that SCRs in response to the CS+ were significantly larger than those to the CS- in both block 1 and block 2 (p < 0.05). Moreover, SCRs in response to both CS+ and CS- were significantly larger in block 1 than in block 2 (p < 0.05). No other significant main effects or interactions were found (Table 1, Fig. 2 and Supplemetary document Table S2, Fig. S1).

#### Recall phase (Day 3)

The mixed model ANOVA revealed significant main effects of Stimulus (F (1, 42) = 7.06, p = 0.011), Block (F (1, 42) = 17.723, p < 0.001), and a significant interaction of Stimulus × Block × Group (F (1, 42) = 4.374, p = 0.043). Post-hoc analyses revealed that in the sham group, SCRs in response to the CS+ were significantly larger than those to the CS- during block 1 (p = 0.004), while no significant difference was observed in the real group between CS+ and CS- in the same block (p = 0.469). No significant differences appeared between the real and sham groups for either the CS+ or CS- in both block 1 and block 2 (p > 0.05). No other significant main effects or interactions were found (Table 1, Fig. 2 and Supplementary document Table S2, Fig. S1).

We additionally analysed relative SCR differences (CS + - CS-) for all phases. These findings showed similar patterns observed in the absolute SCR measures (Supplementary document Fig. S2; Tables S3a, b).

The results of the other questionnaires (psychological assessments, US rating, PANAS, valence, arousal, fear ratings, tDCS blinding, and tDCS adverse effects) are provided in the Supplementary Document.

### fMRI BOLD activations for early and late blocks of learning

#### Whole-brain activation during acquisition

**Fig. 3:**
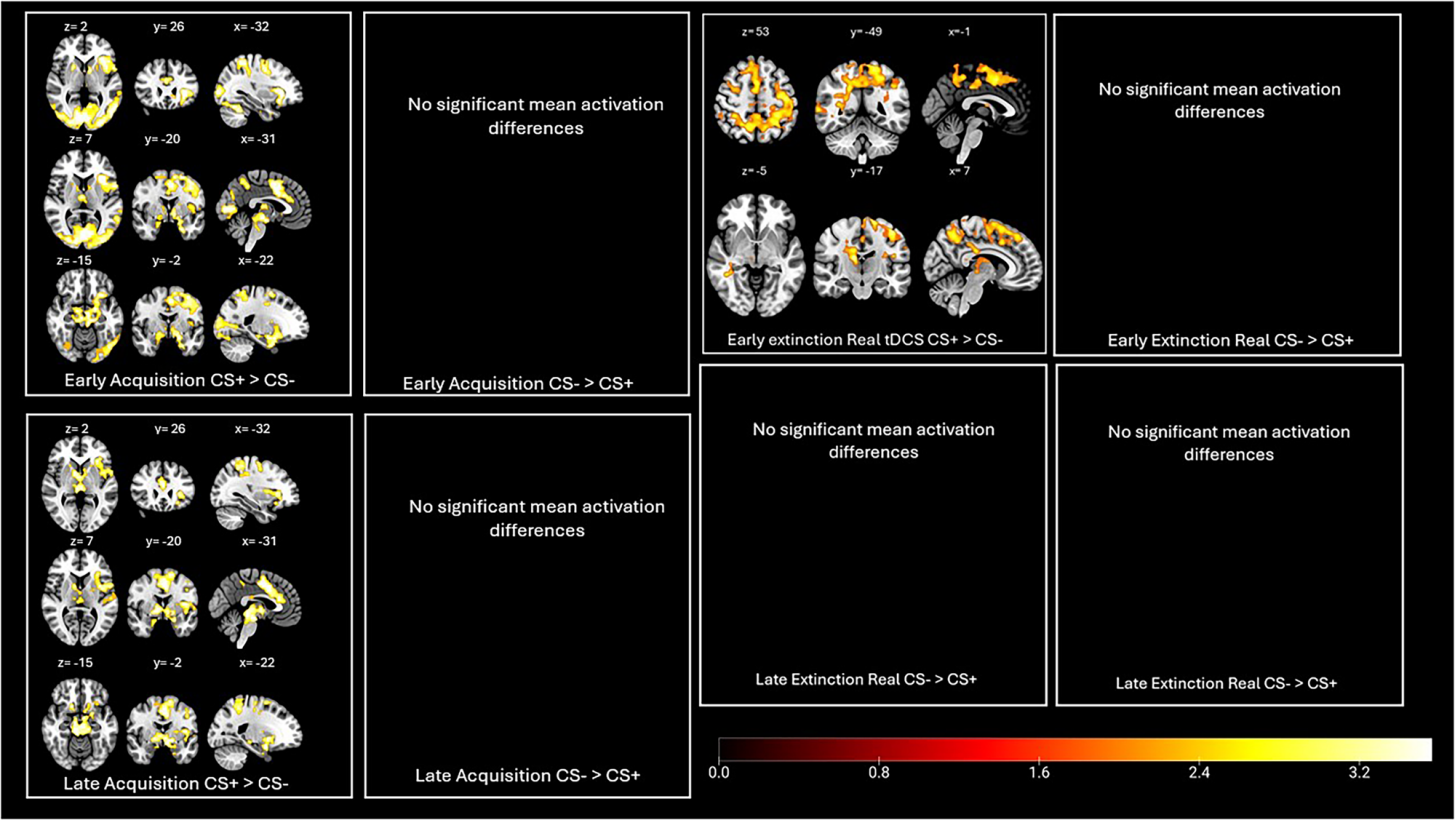
Neural correlates of fear acquisition (left) and fear extinction (right) for real tDCS (thresholded z-maps). No significant activation was observed in the sham tDCS group. Threshold-free cluster enhancement (TFCE) with a p threshold of 0.05 is shown. Anatomical labels were assigned with the Harvard-Oxford cortical and subcortical structural atlas (Harvard - Oxford Cortical Structural Atlas (RRID:SCR_001476)) and the Cerebellar Atlas in the MNI152 space after normalization with FLIRT [70]. Activations are shown for the MNI152 template. The color bar indicates the z-scores ranging from 0 to 3.2, where higher values are associated with larger differences.

Real and sham tDCS groups were analyzed together, as the phase was identical and SCRs did not differ between intervention groups during acquisition. During early and late acquisition, significant activations for the CS+ > CS- contrast were observed in fear network regions, including the left insular cortex, right amygdala, left orbitofrontal cortex, anterior left parahippocampus, left inferior temporal gyrus, brainstem, and right cerebellum (IV, VI, Crus I). Additional clusters included the bilateral paracingulate and anterior cingulate cortices extending to the supplementary motor cortex, bilateral thalamus, left caudate, and left cerebellum (VI, Crus I) (Table 2, Fig. 3). No significant activation was found for the CS- > CS+ contrast.

**Table 2:** CS+ > CS- activations during acquisition, extinction, and recall.

Voxel cluster (TFCE: Threshold Free Cluster Enhancement)	p-FEW (Family-wise error corrected *p*-value)	z-score	Voxel cluster global maxima coordinate in MNI (mm)			Local maxima within the voxel cluster (L: Left, R: Right, and Bi: Bilateral hemispheres)
			**x**	**y**	**z**	
**Early Acquisition: CS+ > CS-**						
12232	0.01	3.54	−32	−54	−34	L. Superior Parietal Lobule, L. Angular Gyrus, L. Occipital Pole, L. Intracalcarine Cortex, L. Lateral Occipital Cortex, L. Lingual Gyrus
9933	0.03	3.54	−44	−10	−36	L. Inferior Temporal Gyrus (anterior and posterior division), L. Temporal Fusiform Cortex (anterior division), R. Amygdala, L. Orbitofrontal Cortex, L. Putamen, L. Insular Cortex, L. Parahippocampal Gyrus (anterior division), Brainstem
263	0.04	3.54	34	−58	−22	R. Cerebellum (IV, VI, Crus I), R. Occipital Fusiform Gyrus
14	0.05	3.54	−20	2	14	L. Cerebral White Matter
Additional regions in the clusters: L. Cerebellum (VI, Crus I), Bi. Paracingulate Gyrus, Anterior Cingulate Cortex, Supplementary Motor Cortex, L. Insular Cortex, L. Frontal Operculum, Bi. Nucleus Accumbens, L. Caudate, Bi. Thalamus						
**Early Acquisition: CS- > CS**+						
no significant mean activation differences						
**Late Acquisition: CS+ > CS-**						
7690	0.03	3.54	−58	−22	10	L. Middle Temporal Gyrus, L. Superior Parietal Lobule, L. Precentral Gyrus, L. Superior and Middle Frontal Gyrus, L. Supramarginal Gyrus (posterior division), L. Precuneus Cortex, L. Postcentral Gyrus, L. Posterior Cingulate Gyrus
5724	0.03	3.54	18	4	−26	L. Temporal Pole, L. Parahippocampal Gyrus (anterior division), L. Amygdala, L. Precentral Gyrus, L. Insular Cortex, L. Central Opercular Cortex and Orbitofrontal cortex, L. Inferior Frontal Gyrus, L. Thalamus
77	0.05	3.54	36	−57	−23	R. Cerebellum (VI, Crus I)
56	0.05	3.54	0	−15	8	L. Thalamus
18	0.05	3.54	−4	−26	−32	Brainstem
Additional regions in the clusters: L. Supramarginal Gyrus (anterior division), L. Parietal Opercular Cortex, L. Posterior Cingulate Cortex, L. Precuneus, Bi. Paracingulate Gyrus, Bi. Anterior Cingulate Cortex, Bi. Supplementary Motor Cortex, R. Parahippocampus Gyrus (anterior division), R. Amygdala, Bi. Pallidum, Bi. Nucleus Accumbens, R. Thalamus						
**Late Acquisition: CS- > CS**+						
no significant mean activation differences						
**Early Extinction: CS+ > CS- Real tDCS**						
15773	0.01	3.54	24	−42	10	L. Supramarginal Gyrus (posterior division), L. Postcentral Gyrus, L. Precentral Gyrus, L. Parietal Opercular Cortex, L. Central Opercular Cortex, L. Superior Parietal Lobule
22	0.05	2.77	42	12	38	R. Middle Frontal Gyrus
18	0.05	2.95	−44	8	4	L. Central Opercular Cortex, L. Frontal Opercular Cortex
9	0.05	2.6	−58	12	24	L. Inferior Frontal Gyrus
7	0.05	2.65	−66	−22	16	L. Postcentral Gyrus
Additional regions in the clusters: Bi. Posterior Cingulate Cortex, Bi. Anterior Cingulate Cortex, Bi. Supplementary motor Cortex, Bi. Superior Frontal Gyrus, Bi. Paracingulate Cortex, R. Superior Parietal Lobule, Bi. Precuneus Cortex, L. Angular Gyrus, R. Medial Temporal Gyrus						
**Early Extinction: CS+ > CS- Sham tDCS**						
4256	0.01	3.54	−10	−32	−14	Bi. Thalamus, Brainstem, L. Pallidum
3	0.05	2.48	−44	−12	−16	L. Cerebral White Matter
3	0.05	3.24	−36	54	20	L. Frontal Pole
Additional regions in the clusters: L. Cerebellum (I-VI and V), L. Temporal Pole, L. Central Opercular Cortex, L. Orbitofrontal Cortex, L. Caudate, Bi. Putamen, R. Nucleus Accumbens, L. Insular Cortex, L. Parahippocampal Gyrus (posterior and anterior divisions), L. Inferior Frontal Gyrus						
**Early Extinction: CS+ > CS-**						
no significant mean activation differences for Real > Sham tDCS and Sham > Real tDCS						
**Early Extinction: CS- > CS**+						
no significant mean activation differences for Real tDCS, Sham tDCS, Real > Sham tDCS, and Sham > Real tDCS						
**Late Extinction: CS+ > CS- Real tDCS**						
no significant mean activation differences						
**Late Extinction: CS+ > CS- Sham tDCS**						
236	0.03	2.99	56	10	−8	R. Temporal Pole, R. Insular Cortex, R. Central Opercular Cortex
**Late Extinction: CS+ > CS-**						
no significant mean activation differences for Real tDCS, Sham tDCS, Real > Sham tDCS, and Sham > Real tDCS						
**Late Extinction: CS- > CS**+						
no significant mean activation differences for Real tDCS, Sham tDCS, Real > Sham tDCS, and Sham > Real tDCS						
**Early Recall: CS+ > CS-**						
no significant mean activation differences for Real tDCS, Sham tDCS, Real > Sham tDCS, and Sham > Real tDCS						
**Early Recall: CS- > CS**+						
no significant mean activation differences for Real tDCS, Sham tDCS, Real > Sham tDCS, and Sham > Real tDCS						
**Late Recall: CS+ > CS-**						
no significant mean activation differences for Real tDCS, Sham tDCS, Real > Sham tDCS, and Sham > Real tDCS						
**Late Recall: CS-** > **CS**+						
no significant mean activation differences for Real tDCS, Sham tDCS, Real > Sham tDCS, and Sham > Real tDCS						

The clusters are reported for the early and late blocks of the experimental phases. All significant clusters are displayed along with regions in the clusters where local maxima were reported in MNI coordinates. For the larger clusters, additional regions beyond the local maxima that exhibit significant activation are also reported.

#### Whole-brain activation during extinction

No significant mean activation differences were found between the group comparison of real and sham tDCS for CS+ > CS− or CS − > CS+ contrasts. During early extinction, real tDCS resulted in significant activation for the CS+ > CS- contrast in both fear and safety networks. The safety network regions included the right middle and medial temporal gyri, bilateral posterior cingulate gyrus, and bilateral precuneus (Table 2, Fig. 3). Sham tDCS showed significant activation selectively in fear network regions, including bilateral thalamus, brainstem, left cerebellum (I–VI, V), left orbitofrontal cortex, and left insular cortex (Table 2, Fig. 3).

During late extinction, no significant activations were observed for real tDCS. For sham tDCS, significantly active regions for the CS+ > CS- contrast were the right temporal pole, the right insular cortex, and the central opercular cortex (Table 2, Fig. 3). No significant activation was found for the CS- > CS+ contrast in early and late extinction.

#### ROI analysis of the vmPFC during extinction

In order to test an active modulation of the vmPFC by tDCS a ROI analysis for vmPFC was conducted. This analysis examined differential vmPFC activation for real > sham tDCS during extinction. Results showed that, in the early extinction block, CS+ resulted in significantly larger activation for real tDCS, and during the late block, CS- was associated with significantly larger activation in the case of real tDCS compared to sham tDCS (Supplementary document Fig. S3, S4).

#### Whole-brain activation during recall

No significant mean activation differences for both CS+ > CS- and CS- > CS+ contrasts were observed for real versus sham tDCS and vice versa for either early or late recall blocks.

#### Additional exploratory whole-brain activation analysis

During fear learning, CS+ activates the fear network and CS- the safety network, reflected in CS+ > CS- and CS- > CS+ contrasts. During extinction and recall, CS+ and CS- responses begin to converge, likely due to reduced CS+ responses (as seen in SCR). Therefore, an exploratory analysis was conducted to show CS+ and CS- activations during extinction and recall. The analyses (Supplementary Document) showed key activations in the amygdala and middle frontal gyrus/frontal pole during early and late extinction, with significantly greater activation under real versus sham tDCS for both CS+ (Supplementary document Fig. S5, Table S8) and CS− (Supplementary document Fig. S6, Table S8). During early recall, greater activation in middle temporal and angular gyri (safety network) was observed for real tDCS in both CS+ (Supplementary document Fig. S7, Table S9) and CS− (Supplementary document Fig. S8, Table S9) trials.

### Seed-based (vmPFC) functional connectivity during extinction for the early and late block of CS+ > CS- for real < sham tDCS

**Fig. 4:**
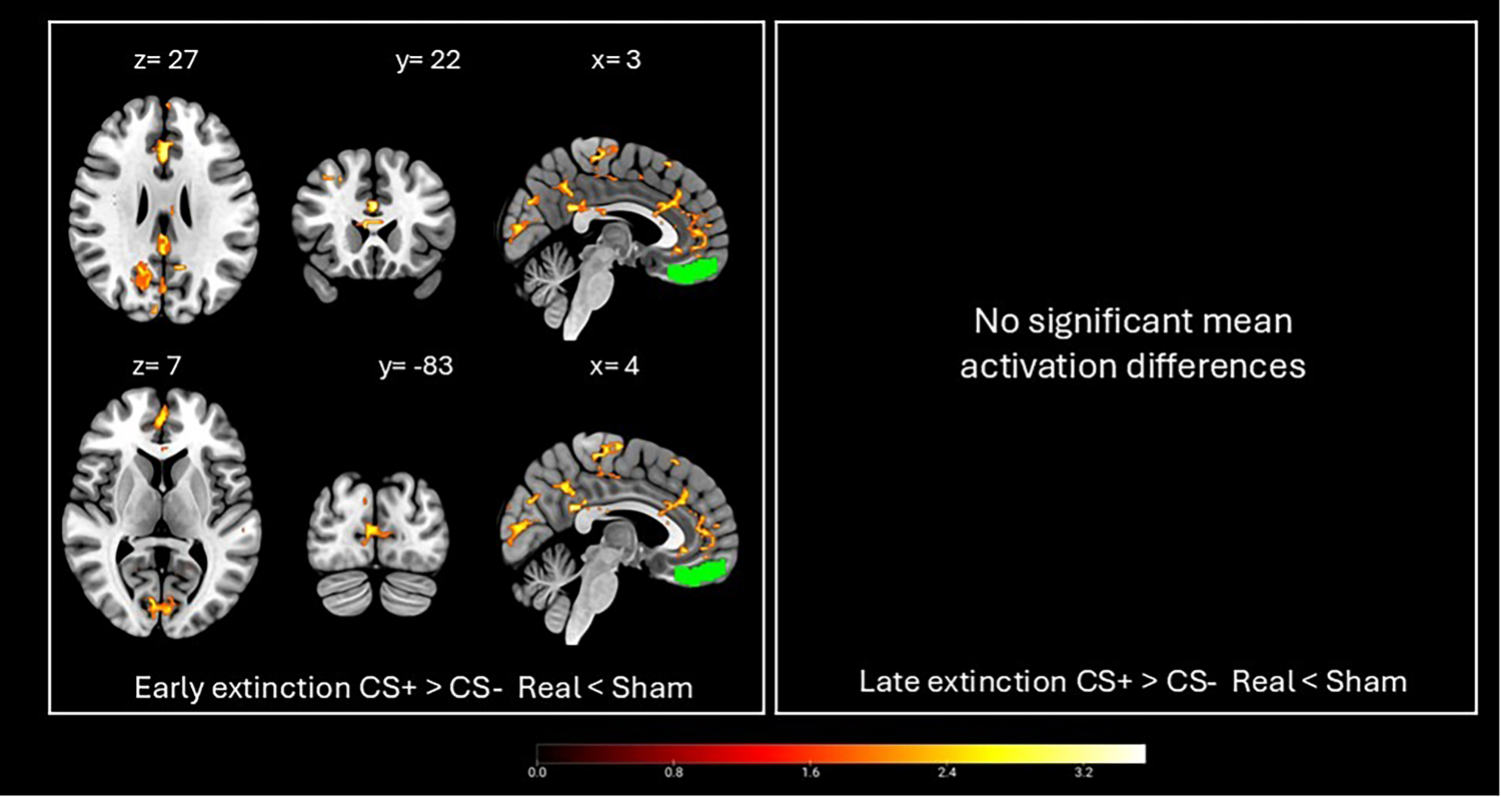
CS+ > CS- seed-based functional connectivity analysis during extinction for real versus sham tDCS (thresholded z-maps). The bilateral vmPFC was used as the seed for this analysis (seed mask shown in green color). Threshold**-**free cluster enhancement (TFCE) with a p threshold of 0.05. Anatomical labels were assigned according to the Harvard-Oxford cortical and subcortical structural atlas (Harvard - Oxford Cortical Structural Atlas (RRID:SCR_001476)) and Cerebellar Altas in MNI152 space after normalization with FLIRT. Connectivity results are displayed in the MNI152 brain. The color bar indicates the z-scores ranging from 0 to 3.2, where higher values are associated with larger differences.

During the early extinction block, compared to sham tDCS, real tDCS showed significantly stronger functional decoupling for CS+ >CS- between the vmPFC and bilateral anterior (ACC) and posterior (PCC) cingulate gyrus, right middle and superior frontal gyrus, right pre- and post-central gyrus, and left superior frontal gyrus (Table 3 and Fig. 4). These results are replicated in the CS+ and CS- analyses (Supplementary document Fig. S10, Table S10). These results, together with the vmPFC ROI analysis, provide further support for an active modulation of the vmPFC by tDCS.

**Table 3:** CS+ > CS- seed-based functional connectivity analysis during extinction for real versus sham tDCS.

Voxel Cluster (TFCE: Threshold Free Cluster Enhancement)	p-FEW (Family-Wise Error Corrected *P*-Value)	z-score	Voxel Cluster Global Maxima Coordinate in MNI (mm)			Local Maxima within the voxel cluster (L: Left, R: Right, and Bi: Bilateral Hemispheres)
		***X***	***Y***	***Z***		
**Early Extinction: CS+ > CS- Real TDCS < Sham tDCS**						
1111	0.039	3.54	2	−20	20	R. Precentral Gyrus, Bi. Posterior Cingulate Gyrus, R. Postcentral Gyrus, L. Paracingulate Gyrus, L. Anterior Cingulate Gyrus
802	0.040	3.54	−4	36	−10	Bi. Anterior Cingulate Gyrus, Bi. Paracingulate Gyrus, Bi. Superior Frontal Gyrus, L. Lingual Gyrus
237	0.030	3.54	−14	−54	−2	L. Precuneus, R. Middle Frontal Gyrus
109	0.029	3.54	32	10	38	R. Middle Frontal, R. Precentral Gyrus
39	0.047	2.99	42	−20	56	R. Postcentral Gyrus
33	0.036	3.54	−4	14	40	L. Anterior Cingulate Gyrus, L. Paracingulate Gyrus
27	0.047	3.54	32	38	42	R. Middle Frontal Gyrus
27	0.047	3.35	2	28	58	R. Superior Frontal Gyrus
3	0.047	3.35	−58	−28	4	L. Superior Temporal Gyrus (posterior division)
**Late Extinction: CS+ > CS- Real TDCS < Sham tDCS**						
no significant mean activation differences						
**Early Extinction: CS- > CS+ Real TDCS < Sham tDCS**						
no significant mean activation differences						
**Late Extinction: CS- > CS+ Real TDCS < Sham tDCS**						
no significant mean activation differences						

Clusters are reported for the early and late blocks. All significant clusters are displayed along with regions in the clusters; local maxima are reported in MNI coordinates.

## Discussion

In the current study, fear-conditioned healthy participants received either anodal (real) or sham tDCS over the vmPFC during extinction learning. Real tDCS led to significantly improved extinction recall in early trials. This was reflected by a non-significant CS+ and CS- SCR difference due to a reduced CS+ amplitude from acquisition to extinction, suggesting a diminished CS + US association and reduced fear responses.

Fear acquisition activated regions of the fear network, including the ACC, amygdala, OFC, and insula. During extinction, distinct activations were observed within real and sham groups, though no group differences reached significance. Furthermore, the ROI analysis revealed greater vmPFC activation in the real tDCS group, which also showed co-activation of safety network regions (e.g., PCC, middle temporal cortex) alongside fear network regions, particularly in early extinction. Finally, seed-based PPI analysis indicated stronger functional decoupling between vmPFC and fear network regions with real tDCS, consistent with an inhibitory vmPFC effect on the fear network, thereby suppressing fear responses.

Unlike prior studies, this study applied tDCS bilaterally over the vmPFC using a nasion electrode, as both hemispheres are critical for emotion regulation and anxiety disorders [17–21, 40]. Anodal tDCS during extinction learning in our study did not enhance immediate extinction but affected recall, in contrast to studies reporting facilitation of extinction learning [33, 35, 36]. Such discrepancies likely stem from differences in task design (timing, duration, extinction/recall structure) and key tDCS parameters (stimulation site, electrode size, intensity, and duration), which significantly affect intervention outcomes [38, 39]. Abend et al. [36] used a 3-day design with larger electrodes over the medial PFC (1.5 mA, 20 min) and found anxiety generalization, likely due to current spread to neighboring regions caused by the larger electrode size. In contrast, Dittert et al. [35] applied 1.5 mA anodal tDCS for 20 min over F7/F8 in a 2-day design (extinction immediately after acquisition) and showed improved extinction in the real tDCS compared to sham. However, a key limitation of this finding is that the faster loss of CS+/CS− discrimination was driven not only by a reduced CS+ response, but also by an unexpected initial increase in responses to the CS−, suggesting a potential disruption of fear-learning consolidation. In a similar 2-day study design, van’t Wout et al. [33] stimulated AF3 and observed that tDCS administered during 2nd extinction block promoted late extinction learning compared with a-tDCS during 1st extinction block), likely due to an anxiolytic after-effect of tDCS. Differences in extinction timing relative to fear learning might be crucial [64]. Unlike the current study, in 2-day studies, immediate extinction may disrupt fear consolidation rather than form an extinction memory [64]. Animal studies showed that blocking protein synthesis immediately after fear conditioning impaired fear memory consolidation and reduced conditioned fear responses [72, 73], and immediate extinction after fear learning did not engage the medial prefrontal cortical circuits crucial in extinction learning [74, 75], suggesting that a 3-day paradigm is a better model of exposure therapy to induce extinction.

Few studies explored tDCS effects during recall, an important phase to show maintenance and effectiveness of extinction learning. Vicario et al. [34] reported improved extinction and recall with 2 mA, 10 min tDCS over the AF3 electrode position during extinction in a 2-day design, whereas van’t Wout et al. [33] found no effects on recall with similar parameters. In PTSD patients, van’t Wout et al. [76] observed that real tDCS administered post-extinction improved early recall compared to stimulation during extinction, likely by facilitating extinction consolidation. Unlike Vicario et al. [34], our findings showed extinction improvement by tDCS, as monitored by SCR only during recall, not extinction, possibly because extinction did not immediately follow acquisition. The extinction effect in Vicario et al. may thus be caused by the specific timing of extinction, since conducting extinction shortly after acquisition could weaken fear memory [64].

In the present study, tDCS effects were observed during extinction recall rather than during extinction learning. This suggests that vmPFC stimulation may preferentially have influenced extinction memory processes beyond online learning and encoding, namely consolidation. Specifically, the stronger effects at recall rather than during extinction learning are consistent with an impact on extinction memory consolidation. Future studies varying stimulation timing relative to learning and consolidation will be needed to clarify the stages of extinction memory most sensitive to vmPFC modulation. For this purpose, beyond tDCS during extinction learning, stimulation could be applied immediately after extinction to enhance post-encoding consolidation, a few hours later to further strengthen consolidation, or during recall to directly facilitate retrieval. Another promising approach is sleep-dependent stimulation, particularly during REM sleep, a phase critical for the consolidation and stabilization of extinction memories [77, 78]. Comparing these conditions would help to determine which stage, from learning to recall, is most sensitive to vmPFC modulation.

Future translational work should evaluate the study protocol efficacy in high-risk (subclinical) and patient groups with impaired extinction learning. These studies should further aim to optimize stimulation dosing and determine the optimal timing of this intervention relative to extinction learning and consolidation by comparing tDCS applied during initial extinction learning versus during the consolidation phase. Additionally, combining tDCS with standardized therapy-based interventions, while assessing both clinical outcomes and mechanistic markers of extinction, will be key to establishing the therapeutic utility of tDCS, and to identifying the patients with the most likely benefit.

Moreover, translating these findings to patients who often have long-standing fear memories and extended exposure histories may require multiple extinction and/or tDCS sessions. Protocols may also need to be tailored to therapy structure, such as imaginal versus in-vivo exposure, to optimally engage extinction-related networks and improve clinical outcomes. Future studies varying stimulation timing relative to extinction learning and consolidation will be essential to clarify the stages of extinction memory most sensitive to vmPFC modulation.

### MRI activations during fear acquisition and extinction learning

Successful fear learning in our study is supported by the activation of key fear network regions [9, 79], aligning with results of respective meta-analyses [10–12]. This includes activation of the ACC, insular cortex, amygdala, cerebellum, and supplementary motor areas involved in fear learning. Among these, the ACC and insular cortex are integral to the central autonomic–interoceptive network, with the insular cortex integrating cognitive, affective, and physical states, while the ACC facilitates homeostatic responses [80, 81]. Their coactivation is linked to autonomic arousal [82] and is consistently reported in fear-learning studies [10–12]. Activation of the brainstem, thalamus, and parahippocampal gyrus during fear learning in the current study indicates their role in viscerosensory information relay [83]. Furthermore, amygdala activation, which regulates top-down fear expression, was observed [6, 15].

In the late fear learning phase, activation was also observed in the bilateral amygdala, parahippocampal gyrus, left precentral and postcentral gyri, left precuneus, and posterior cingulate gyrus. This broader activation of fear and default mode network (DMN) regions, including the precuneus and posterior cingulate, suggests an integration of complex brain networks [84].

Consistent with previous studies, we did not observe any significant activation in the safety network (CS- > CS + ) during the fear acquisition phase [85, 86]. However, a few studies, including a meta-analysis, have reported safety network activation during this phase [14, 83, 84]. Considering that research on the safety network is limited and findings remain inconsistent, we checked the fear acquisition data using a more liberal threshold (p-threshold of 0.25 rather than 0.05) [85, 86]. At this threshold, significant activation of the safety network was observed during the early block, with activations of the vmPFC, PCC, precentral gyrus, and OFC (Supplementary document Fig. S9). These results reflect that safety signalling was not sufficiently robust and consistent for generating CS- > CS+ effects across our participants.

Significant brain activations were observed for early and late extinction phases, respectively, within the real and sham tDCS groups. During early extinction, both sham and real tDCS groups showed significant activations for the CS+ versus CS- contrasts. In the sham tDCS group, major fear network regions, including the insular cortex, orbitofrontal cortex, thalamus, left parahippocampal gyrus, brainstem, and left frontal pole were active, consistent with previous studies [87, 88]. In contrast, the real tDCS group showed activations in fear network regions and in DMN regions, including the posterior cingulate gyrus, precuneus, inferior frontal gyrus, and medial frontal gyrus [89]. These DMN network regions are part of the safety network, as shown in fear-learning studies [11]. The additional recruitment of regions linked to safety processing in the real tDCS group may indicate concurrent engagement of fear- and safety-related networks during extinction. This pattern is consistent with the possibility that vmPFC stimulation modulated processes related to contextual appraisal, emotion regulation and safety processing. Such integration may underlie the observed facilitation of extinction learning and improved recall with real tDCS.

Interestingly, similar to our previous tDCS findings, where modulation of the left inferior frontal gyrus (IFG) via tDCS suggested an involvement of this region in extinction [62], the current study also observed activation of that area in both real and sham tDCS groups during the early extinction phase. This region contributes to emotional processing and shows heightened responses to threat cues compared to safety cues in fear extinction studies [90, 91],which may explain its coactivation with other fear network regions during extinction in the current study. While Ma et al. [62] showed that anodal tDCS over the IFG reduced extinction and cathodal tDCS enhanced it, suggesting polarity-dependent effects of direct tDCS over the IFG on extinction, our results suggest that indirect IFG activation via vmPFC stimulation may reflect functional recruitment of a broader extinction-related network, highlighting the importance of stimulation site and network-level dynamics in shaping tDCS effects on fear extinction.

During early extinction, temporal regions - the temporal pole and the middle temporal gyrus -were activated in sham and real tDCS conditions. These regions connect to the amygdala and may influence emotional memory. The temporal pole is involved in social and emotional processing [92] and its enhanced connectivity with the amygdala may contribute to hyperactivity in fear network, relevant to anxiety disorders [93].

During late extinction, the insular cortex and temporal pole were active in the sham tDCS group, but no significant activations were found in the real tDCS group. This absence of activation in the real tDCS group was likely due to the similar neural CS+ and CS- responses, resulting in no meaningful differential activation in the CS+ > CS- contrast. Activation in the sham group may indicate incomplete extinction, which could explain reduced extinction recall for the sham group.

Unlike previous extinction learning studies, when comparing the CS+ > CS- contrast, we did not observe an involvement of the amygdala [6] or vmPFC in the extinction phase [94] in either the real or sham tDCS groups, consistent with meta-analyses showing no significant activation of these regions [87]. However, this does not necessarily imply that these regions are not involved in extinction but may be due to comparable CS+ and CS- activation levels in the amygdala and vmPFC. Supporting this, our exploratory analyses showed significant amygdala and medial frontal/frontal pole activation during early and late extinction for CS+ and CS- in real > sham tDCS (Supplementary document, Figures S5–S6, Table S8). Also, the ROI analysis of the vmPFC revealed greater early CS+ activation and greater late CS− activation for real tDCS compared to sham, highlighting its dynamic role in processing both CS+ and CS- stimuli (Supplementary document Fig. S4).

In contrast to other studies, no mean activation differences were found for CS+ versus CS- in the recall phase. The studies showing activations during recall were either meta-analyses [87], used vmPFC as the region of interest for analysis [6], or re-exposed the participants to the US after extinction [89]. Furthermore, the missing activation difference of the CS+ versus CS- in our study could be due to the convergence of CS+ and CS- responses, likely due to reduced CS+ responses (as seen in the SCR responses). Interestingly, in our study, it was observed that in the early recall condition for the real tDCS group, the middle temporal gyrus and angular gyrus were active for both the CS+ (Supplementary document Fig. S7, Table S9) and CS- (Supplementary document Fig. S8, Table S9) trials, aligning with their role in the safety network [11].

### Functional connectivity during extinction learning

During early extinction, real tDCS (vs. sham) produced stronger functional decoupling for CS+ > CS- between the vmPFC and bilateral ACC, PCC, right middle/superior frontal gyrus, right pre-/post-central gyrus, and left superior frontal gyrus (Table 3, Fig. 4). These fear and DMN network regions were also active during fear acquisition. Functional decoupling has been linked to suppression of neural responses [51], suggesting that stronger decoupling under real tDCS reflects an inhibitory effect of vmPFC activation on the fear network, thereby reducing fear responses. However, we did not replicate earlier findings that amygdala–medial frontal connectivity is critical for extinction [6, 95], likely due to differences in paradigms and the contrasts used to report the results. Milad et. al used a 2-day paradigm with 2 different types of CS+ and one type of CS-. During extinction (followed immediately after acquisition), only one type of CS+ was included, and during recall, both types of CS+ and the CS- were presented. vmPFC-amygdala connectivity was observed during the recall phase for the contrast between the extinguished CS+ versus the non-extinguished CS+. In another study, Ganella et al. tested acquisition, extinction, and recall on the same day, and the participants were reconditioned for the CS+ and CS- before recall. Interestingly, they observed a significant negative vmPFC-amygdala connectivity during recall for the contrast CS+ versus CS-. They interpreted this finding as suggesting a top-down inhibitory effect of the PFC on amygdala reactivity.

#### Limitations and future directions

Several limitations of this study should be considered. First, we relied on SCR responses to assess fear learning and extinction. While SCR is the most common measure of conditioned fear, it is sensitive to movement and temperature [58, 96]. Consistent with established practice, participants who did not show differential conditioning during acquisition or whose SCR signals were low or noisy were excluded to ensure reliable discrimination measures. While this approach strengthens the reliability of the SCR index, it may preferentially retain individuals with clearer autonomic discrimination. Given that anxiety disorders are associated with variability of autonomic responsivity and fear-learning profiles, incorporating additional indicators such as pupillary response and heart rate could provide a more comprehensive physiological profile [97] and help to further determine generalizability of the present findings to clinical populations. Second, a direct comparison with other tDCS studies was limited by different experimental design and stimulation parameters. Third, although significant CS+ > CS- activation was observed during acquisition, significant CS- > CS+ differences only appeared at a more liberal threshold (Supplementary document Fig. S9). This suggests that safety network activity was present but weaker and less consistent across participants. Fourth, the study did not include an additional behavioral control group, which could have provided an extra layer of comparison and further clarified the specificity of the observed effects. However, the primary aim was to investigate the mechanistic effects of tDCS on fear extinction, and the sham group served as a rigorous control, accounting for non-specific factors such as participant expectations and task engagement. This approach is consistent with prior tDCS studies of fear learning and extinction, in which sham stimulation typically serves as the sole control condition [33–35]. Fifth, although the Chi-square test indicated successful blinding, the small sample size may have limited power to detect subtle differences, so incomplete blinding cannot be ruled out. Finally, results are preliminary, and replication with larger samples, alternative designs, and clinical groups (e.g., specific phobias) would improve generalizability.

## Conclusion

This study provides evidence that anodal tDCS over the vmPFC during extinction learning stabilized extinction memory. SCR measures showed that real tDCS facilitated extinction recall during early trials compared to sham tDCS. Moreover, real tDCS facilitated activation of safety network regions, including the posterior cingulate gyrus and precuneus, alongside the fear network. This engagement may support consolidation of extinction learning and/or subsequent retrieval of safety memory, explaining improved recall. The present study adds important information to another study [98] that showed clinical benefits of multi-session tDCS over the vmPFC. The present study addressed underlying physiological mechanisms and provides direct evidence that tDCS enhances extinction learning via specific neural mechanisms, a key translational link. Given the variability of tDCS effects across individuals and identifying the vmPFC as a mechanistically relevant target highlights its potential for refining and personalizing tDCS treatment protocols. Overall, these findings underscore the translational potential of tDCS as an adjunctive treatment for anxiety disorders and highlight the need for replication in sub-clinical and anxiety populations.

## Supplementary information

Supplementary Document

## Data Availability

The data that support the findings of this study are not openly available due to reasons of sensitivity, and anonymized data are available from the corresponding author upon reasonable request.
