# Supplementary material for: The physiological foundation of extinction improvement by tDCS over the ventromedial prefrontal cortex (vmPFC) in healthy humans: an fMRI study
Source: Transl Psychiatry. 2026 Jun 26;16:319. doi: 10.1038/s41398-026-04190-4 (PMC13309533; doi:10.1038/s41398-026-04190-4)
Supplement: Supplementary file 1 — Supplementary Document [file 41398_2026_4190_MOESM1_ESM.docx]

**Supplementary Document**

1. **Methods**
   1. **Fear conditioning and extinction task**

The three phases of the Pavlovian fear conditioning paradigm, namely fear acquisition, extinction, and extinction recall [[1](#_ENREF_1)] were administered over three consecutive days (approximately 24-hour intervals). The experiment consisted of a neutral context (computer desk table with a desk lamp on the right) with the light color of the lamp (CS+: blue, CS-: yellow) serving as the two conditioned stimuli. The unconditioned stimuli (US) for the fear acquisition phase were aversive electric stimuli paired with the CS+. The CS- was never followed by the US. The acquisition phase had 16 CS- and 16 CS+ trials with 10 CS+ trials followed by the US (reinforcement rate of 62.5 %). Extinction and extinction recall phases each had 8 CS+ and 8 CS- trials, and no US. Each trial started with a 1.2s neutral context presentation, followed by 12s CS+/CS- presentation. In reinforced trials, the US was applied 11.9 s after CS+ onset and co-terminating with the CS+. Intertrial intervals were indicated by a white crosshair, in the middle of the computer monitor, on a grey background and their duration jittered between 19.2s and 22.8s. To avoid dazzle effects, the background of all images (intertrial intervals and trials) was kept identical (Figure 1).

The acquisition phase was completed within 20min, and the extinction and extinction recall phases each lasted for 10min. Trial order within each phase was pseudorandomized with three conditions: i) during the acquisition phase, the first and the two last CS+ trials were followed by the US; ii) no more than two consecutive trials were the same (CS-, CS+, CS+US), and iii) the number of each trial type (CS-, CS+, CS+US) was identical for the first and second part of each phase. The order of trials was the same for all participants.

The task was presented on a computer via the software Presentation (version 20.0, Neurobehavioral System Inc., Berkeley, CA). The synchronization of stimulus presentation with fMRI acquisition was triggered with MRI scan triggers supplied to the control computer. Images were projected onto a MR-compatible screen at the end of the scanner bore. Images were visible to the participants through a mirror mounted on the Simens 64-channel head coil.

The electric stimulation (US) was generated by a constant voltage stimulator (model: STM200, Biopac System Inc., Goleta, CA, United States; intensity: up to 100 V range at 0.1-200ms pulse width) and applied to the right index and middle fingertips via disposable, radio translucent, circular pre-gelled electrodes (model: EL508, Biopac System Inc., Goleta, CA, United States). The electrode position was kept consistent on all days. The US was 100 ms long and consisted of four consecutive 500 µs current pulses with an inter-pulse interval of 33 ms.

- 1. **tDCS montage modelling**

We used computational modelling to determine the optimal stimulation montage for targeting the vmPFC. A search space comprising all possible combinations of two square electrodes at 10-20 EEG positions - representing the traditional and most commonly used tDCS configuration in terms of shape and location -, and various 4×1 electrode configurations - which offers better focality-, was simulated using ROAST (https://www.parralab.org/roast/) [2]. For each montage, the average electric field (EF) was calculated in the left dlPFC, right dlPFC, vmPFC, ACC, and cerebellum. The final montage for targeting the vmPFC was selected based on the maximum average EF in the target region and minimum average EF values in the other above-mentioned regions (five round electrodes, 2 cm diameter each, one at the nasion and four at F7, F8, Ex19, and Ex20, following the 10–5 EEG system [[2](#_ENREF_2)] (Fig. 1). Additionally, a post-hoc electric field simulation was done using SimNIBS 4.5 [[3](#_ENREF_3)] on individual anatomy for all participants in the real tDCS group. This was done to get an estimate of the average electric field in the real tDCS group for the bilateral dlPFC, vmPFC and bilateral amygdala (Table S7).

- 1. **Questionnaires**

Participants were required to answer three questionnaires, one before and two after each experimental phase. The emotional state before the task was assessed with the Positive and Negative Affect Schedule (PANAS) [[4](#_ENREF_4)]. Positive and negative affects were scored on a 5-point Likert scale (1- very slightly to 5- extremely) for 10 items each. Following each of the experimental phases and inside the MRI scanner the participants had to rate valence, emotional arousal, fear, and US expectancy for CS+ and CS- trials. All ratings were scored on a 9-point Likert scale from “very unpleasant” to “very pleasant”, “calm and relaxed” to “very excited”, “not afraid” to “very afraid” and “US not expected” to “US surely expected”, respectively. Only post-acquisition, participants were asked to rate US unpleasantness (9-point Likert scale “not unpleasant” to “very unpleasant”) and the percentage of US presentations with CS presentations.

tDCS blinding and adverse effects were assessed by administering a well-introduced adverse-effects questionnaire before and after the fMRI sessions [[5](#_ENREF_5)]. After each session, participants had to answer if they received tDCS or not and had to rate on a 6-point Likert scale (“no sensation” to “very strong”) if they felt any sensations, namely itching, tingling, burning, or pain. 24 hours following each session, participants had to rate on a 6-point Likert scale (“no sensation” to “very strong sensation”) the presence of skin redness, headache, fatigue, concentration difficulties, nervousness, and sleep problems.

- 1. **Questionnaires Statistical Analysis**

All statistical analyses were conducted with IBM SPSS Statistics software (IBM Corp., IBM SPSS Statistics for Windows, Version 26.0. Armonk, NY, United States). Chi-square tests and independent sample t-tests were conducted to evaluate tDCS blinding efficacy, and the tDCS adverse effects profile. The PANAS was analyzed via a mixed model ANOVA, with Phases (acquisition, extinction, and recall) as within-subject factors, and tDCS (real and sham) as between-subject factor. Fear conditioning and extinction task-induced valence, emotional arousal, fear, and US expectancy for CS+ and CS- trials were analyzed via a mixed model ANOVA, with Phases (acquisition, extinction, and recall), and CS (CS+ and CS-) as within-subject factors, and tDCS (real and sham) as between-subject factor. Statistical significance was set at p ≤ 0.05 for all tests. For all ANOVAs, the Greenhouse–Geisser correction was applied whenever Mauchly’s sphericity test indicated a violation of sphericity. For significant ANOVA results, Fisher’s Least Significant Difference (LSD) post hoc tests were conducted, with statistical significance defined as p < 0.05.

1. **Results**

**Table S1.** Demographic data of the participants. Between-group comparisons were conducted using independent sample t-tests for age and psychological assessment variables. For gender, a chi-square test was performed.

| Descriptives | Real tDCS (n=21)  Mean±SD | Sham tDCS (n=23)  Mean±SD | t/χ² | p |
| --- | --- | --- | --- | --- |
| Age | 26.57±4.40 | 24.13±2.53 | 2.01 | 0.05 |
| ASI-3 total | 17.95±13.47 | 15.70±10.17 | 0.631 | 0.532 |
| BEAQ total | 38.81±9.56 | 43.04±11.02 | -1.355 | 0.183 |
| DASS21 Depression | 4.29±2.78 | 4.00±4.86 | 0.236 | 0.814 |
| DASS21 Anxiety | 3.81±4.29 | 4.26±3.68 | -0.376 | 0.709 |
| DASS21 Stress | 5.62±4.27 | 6.70±4.96 | -0.768 | 0.447 |
| US Intensity | 48.84±15.82 | 44.01±22.46 | 0.82 | 0.42 |
| Sex | M:F: 14:7 | M:F=9:14 | 3.34^$^ | 0.07 |

1. **^$^** χ²= Chi-square test, t= independent sample t-test. M= Male; F= Female; DASS21: Depression Anxiety Stress Scale 21; ASI-3: Anxiety Sensitivity Index; BEAQ: Brief Experiential Avoidance Questionnaire; US: Unconditioned stimuli (electric pulse)

The main effect of Stimulus (CS+ vs. CS-) on SCR across each phase


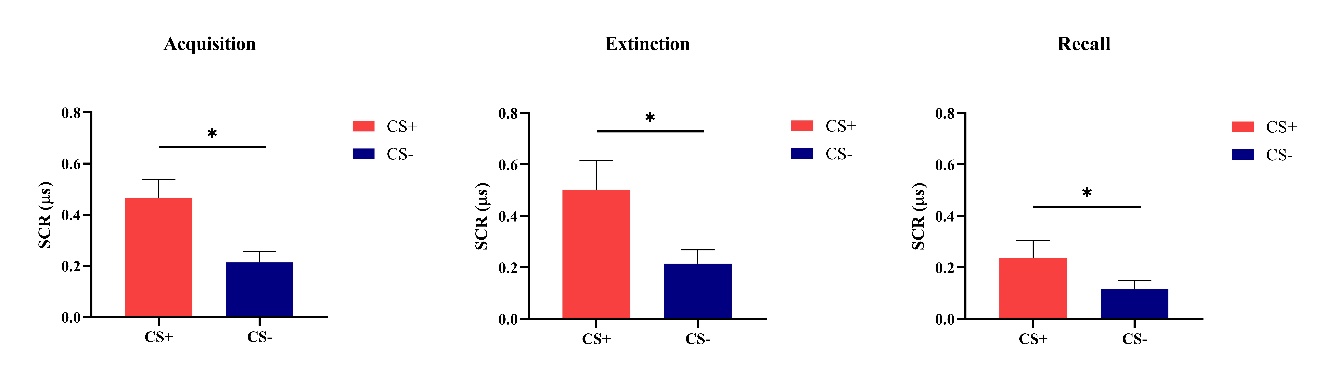


**Figure S1.** Main effect of Stimulus (CS+ vs. CS-) on SCR across each phase. Bar graphs depict the mean SCR (μS) for CS+ (red) and CS- (blue) stimuli during the **acquisition, extinction,** and **recall** phases**.** A significant main effect of Stimulus was observed, with the CS+ eliciting higher SCRs compared to the CS- across all phases (p < .05). Error bars represent the standard error of mean. Asterisks (*) indicate statistically significant differences, p < 0.05.

**Table S2.** Absolute SCR values (µS) of each block for different stimuli across each phase.

|  | | Real tDCS (n=21)  Mean SD | | Sham tDCS (n=23)  Mean SD | |
| --- | --- | --- | --- | --- | --- |
| Block 1 | | | | | |
| Acquisition | CS+ | 0.59 | 0.65 | 0.55 | 0.55 |
|  | CS- | 0.41 | 0.54 | 0.23 | 0.27 |
| Extinction | CS+ | 0.63 | 1.06 | 0.87 | 1.08 |
|  | CS- | 0.35 | 0.56 | 0.28 | 0.42 |
| Recall | CS+ | 0.31 | 0.44 | 0.44 | 0.78 |
|  | CS- | 0.24 | 0.46 | 0.16 | 0.26 |
| Block 2 | | | | | |
| Acquisition | CS+ | 0.39 | 0.46 | 0.33 | 0.42 |
|  | CS- | 0.17 | 0.21 | 0.06 | 0.11 |
| Extinction | CS+ | 0.2 | 0.5 | 0.31 | 0.54 |
|  | CS- | 0.09 | 0.24 | 0.13 | 0.22 |
| Recall | CS+ | 0.1 | 0.27 | 0.09 | 0.31 |
|  | CS- | 0.03 | 0.08 | 0.03 | 0.07 |

**Relative SCR Difference (CS+ - CS-)**

**Acquisition:** The mixed model ANOVA showed no significant main effects or interactions (Table S3a,b and Fig. S2).

**Extinction:** The mixed model ANOVA revealed a significant main effect of Block (F (1, 42) = 7.783, p = 0.008). No other significant main effects or interactions were found (Table S3a,b and Fig. S2).

**Recall:** The mixed model ANOVA revealed a significant Block × Group interaction (F (1, 42) = 4.374, p = 0.043). Post-hoc tests showed that the sham group had a significantly larger SCR difference in block 1 compared to block 2 (p = 0.006). No other significant main effects or interactions were found (Table S3a,b and Fig. S2).


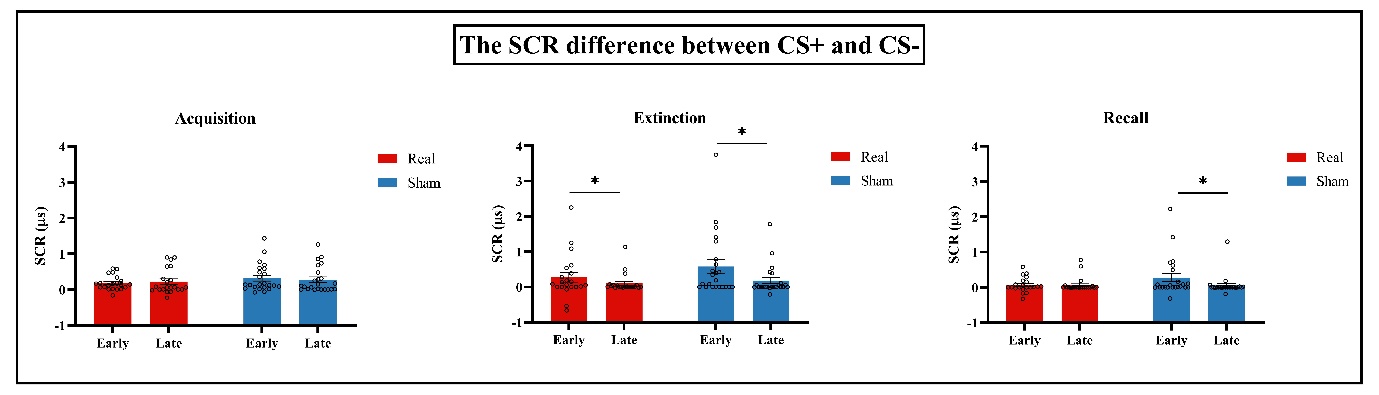


**Figure S2.**  Bar colors represent different groups, and error bars indicate the standard error of mean. During the extinction phase, there was a significant main effect of block, the post hoc tests revealed that both tDCS groups had a significantly larger SCR difference in the early block compared to the late block. During the recall phase, the Group × Block interaction was significant, the sham group showed a significantly larger SCR difference in the early block than the late block. Asterisks indicate significant results of post-hoc comparisons of with respect to block, p < 0.05.

**Table S3a.** Differential SCR values (CS+ minus CS-) of each block across each phase.

|  | | Real tDCS (n=21)  Mean SD | | Sham tDCS (n=23)  Mean SD | |
| --- | --- | --- | --- | --- | --- |
| Acquisition | Block 1 | 0.18 | 0.2 | 0.32 | 0.38 |
|  | Block 2 | 0.22 | 0.34 | 0.28 | 0.36 |
| Extinction | Block 1 | 0.28 | 0.61 | 0.58 | 0.88 |
|  | Block 2 | 0.11 | 0.26 | 0.18 | 0.42 |
| Recall | Block 1 | 0.07 | 0.2 | 0.28 | 0.55 |
|  | Block 2 | 0.08 | 0.2 | 0.06 | 0.27 |

**Table S3b.** Results of the mixed model ANOVAs for assessing the SCR differences between CS+ and CS- presentation across acquisition, extinction, recall phases.

|  | **Factors** | **F value** | **df, error** | **p** | **η_p_^2^** |
| --- | --- | --- | --- | --- | --- |
| **Acquisition** | Block | 0.001 | 1, 42 | 0.97 | 0.00 |
|  | Group | 1.35 | 1, 42 | 0.25 | 0.03 |
|  | Block × Group | 0.55 | 1, 42 | 0.46 | 0.01 |
| **Extinction** | Block | 7.78 | 1, 42 | **0.008** | 0.16 |
|  | Group | 1.54 | 1, 42 | 0.22 | 0.04 |
|  | Block × Group | 1.27 | 1, 42 | 0.27 | 0.03 |
| **Recall** | Block | 3.73 | 1, 42 | 0.06 | 0.08 |
|  | Group | 1.11 | 1, 42 | 0.29 | 0.03 |
|  | Block × Group | 4.37 | 1, 42 | **0.04** | 0.09 |

df: degrees of freedom

**Positive and Negative Affect Scale (PANAS):** The mixed model ANOVA showed significant main effects of phases (F (2,84) = 3.55, p = 0.03), PANAS (F (1,42) = 12.94, p = 0.001), and a significant Phases x PANAS interaction (F (2,84) = 5.38, p = 0.01). The post-hoc tests showed greater overall PANAS scores for the extinction day compared to the recall day (p=0.004), overall negative scores were larger than positive scores (p=0.001), and for both acquisition and recall phases, negative scores were larger than positive scores (p<0.01) (Table S4a, S4b).

**Table S4a.** The PANAS scores for positive and negative affects in each condition

|  |  | Real tDCS (n=21)  Mean SD | | Sham tDCS (n=23)  Mean SD | |
| --- | --- | --- | --- | --- | --- |
| Acquisition | Positive Score | 20.29 | 5.29 | 18.78 | 3.12 |
|  | Negative Score | 22.33 | 5.72 | 20.57 | 4.86 |
| Extinction | Positive Score | 20.86 | 4.64 | 20.70 | 5.79 |
|  | Negative Score | 22.05 | 4.17 | 19.91 | 4.94 |
| Recall | Positive Score | 19.24 | 3.95 | 17.61 | 3.74 |
|  | Negative Score | 21.29 | 4.21 | 19.57 | 4.76 |

**Table S4b.** The results of the mixed model ANOVA for the Positive and Negative Affect Scale.

|  | F | df^*^, error | p | ŋ² |
| --- | --- | --- | --- | --- |
| Phases | 3.55 | 2, 84 | **0.03** | 0.08 |
| PANAS | 12.94 | 1, 42 | **0.001** | 0.23 |
| tDCS | 2.14 | 1,42 | 0.15 | 0.05 |
| Phases * tDCS | 0.13 | 2, 84 | 0.874 | 0.00 |
| PANAS * tDCS | 1.03 | 1, 42 | 0.31 | 0.02 |
| Phases * PANAS | 5.38 | 2, 84 | **0.01** | 0.11 |
| Phases * PANAS * tDCS | 1.41 | 2, 84 | 0.25 | 0.03 |

*df: degrees of freedom

**tDCS blinding efficacy and side-effects:** The chi-square test showed no significant difference between the groups for the guessed real and sham ratings for any of the phases. The results show effective blinding during acquisition (χ ^2^ = 0.30, p = 0.59), extinction (χ ^2^ = 1.37, p = 0.24), and recall (χ ^2^ = 0.63, p = 0.43) phases (Table S5a). Further, the independent samples t-tests showed no significant difference in the adverse effect ratings experienced during tDCS except during the extinction phase. Real tDCS groups reported a significantly greater tingling sensation than the sham tDCS group (t=2.41, p= 0.02) (Table S5b). Lastly, the independent samples t-tests showed no significant difference in the adverse effect ratings experienced after tDCS for real and sham tDCS groups (Table S5c).

**Table S5a.** Chi-square results for tDCS Blinding for all three phases

| Phases | Real (Yes:No) | Sham (Yes:No) | Χ² | p |
| --- | --- | --- | --- | --- |
| Acquisition | 04:17 | 03:20 | 0.30 | 0.59 |
| Extinction | 09:12 | 06:17 | 1.37 | 0.24 |
| Recall | 05:16 | 08:15 | 0.63 | 0.43 |

**Table S5b.** The results of the independent samples t-tests conducted for the adverse effect ratings during tDCS. Rating conducted immediately after tDCS

| Study Phases |  | Real tDCS (n=21)  Mean SD | | Sham tDCS (n=23)  Mean SD | | t | p |
| --- | --- | --- | --- | --- | --- | --- | --- |
| Acquisition | Itching | 0.14 | 0.48 | 0.04 | 0.21 | 0.91 | 0.37 |
|  | Tingling | 0.29 | 0.78 | 0.35 | 0.93 | -0.24 | 0.81 |
|  | Burning | 0.38 | 1.24 | 0.00 | 0.00 | 1.40 | 0.18 |
|  | Pain | 0.14 | 0.65 | 0.04 | 0.21 | 0.69 | 0.49 |
|  |  |  |  |  |  |  |  |
| Extinction | Itching | 0.38 | 0.92 | 0.04 | 0.21 | 1.64 | 0.11 |
|  | Tingling | 0.90 | 1.14 | 0.22 | 0.67 | 2.41 | **0.02** |
|  | Burning | 0.95 | 1.60 | 0.48 | 1.12 | 1.14 | 0.25 |
|  | Pain | 0.33 | 0.66 | 0.30 | 0.88 | 0.12 | 0.90 |
|  |  |  |  |  |  |  |  |
| Recall | Itching | 0.10 | 0.30 | 0.00 | 0.00 | 1.45 | 0.16 |
|  | Tingling | 0.10 | 0.30 | 0.43 | 0.84 | -1.81 | 0.08 |
|  | Burning | 0.19 | 0.68 | 0.22 | 0.85 | -0.12 | 0.91 |
|  | Pain | 0.10 | 0.30 | 0.17 | 0.65 | -0.51 | 0.61 |

**Table S5c.** The results of the independent samples t-tests conducted for the adverse effect ratings 24h after tDCS. Ratings conducted 24 hours after tDCS

|  |  | Real tDCS (n=21)  Mean SD | | Sham tDCS (n=23)  Mean SD | | t | p |
| --- | --- | --- | --- | --- | --- | --- | --- |
| Acquisition | Redness | 0.00 | 0.00 | 0.43 | 1.31 | -1.59 | 0.13 |
|  | Head pain | 0.19 | 0.40 | 0.17 | 0.65 | 0.10 | 0.92 |
|  | Fatigue | 0.29 | 0.78 | 0.39 | 1.03 | -0.38 | 0.71 |
|  | Concentration | 0.05 | 0.22 | 0.13 | 0.63 | -0.58 | 0.57 |
|  | Nervousness | 0.14 | 0.65 | 0.00 | 0.00 | 1.00 | 0.33 |
|  | Sleep Disturbance | 0.00 | 0.00 | 0.00 | 0.00 |  |  |
|  |  |  |  |  |  |  |  |
| Extinction | Redness | 0.19 | 0.51 | 0.17 | 0.65 | 0.09 | 0.93 |
|  | Head pain | 0.24 | 0.89 | 0.17 | 0.49 | 0.30 | 0.77 |
|  | Fatigue | 0.81 | 1.36 | 0.30 | 1.02 | 1.38 | 0.18 |
|  | Concentration | 0.52 | 0.87 | 0.09 | 0.42 | 2.09 | 0.06 |
|  | Nervousness | 0.19 | 0.51 | 0.00 | 0.00 | 1.71 | 0.10 |
|  | Sleep Disturbance | 0.05 | 0.22 | 0.00 | 0.00 | 1.00 | 0.33 |
|  |  |  |  |  |  |  |  |
| Recall | Pain | 0.14 | 0.48 | 0.00 | 0.00 | 1.37 | 0.19 |
|  | Redness | 0.05 | 0.22 | 0.13 | 0.46 | -0.75 | 0.46 |
|  | Head pain | 0.29 | 0.78 | 0.17 | 0.83 | 0.46 | 0.65 |
|  | Fatigue | 0.19 | 0.68 | 0.00 | 0.00 | 1.35 | 0.19 |
|  | Concentration | 0.19 | 0.68 | 0.00 | 0.00 | 1.35 | 0.19 |
|  | Nervousness | 0.14 | 0.48 | 0.00 | 0.00 | 1.37 | 0.19 |

**Valence, arousal, fear, and US expectancy ratings:** For valence, arousal, fear, and US expectancy, the respective mixed model ANOVAs were conducted with Phases (acquisition, extinction and recall), and CS (CS+ and CS+) as within-subject factors and tDCS (real and sham) as between-subjects factor. For valence, arousal, fear, and US expectancy, the mixed model ANOVAs showed significant main effects of Phases (valence: F (2,84) = 23.58, p <0.01; arousal: F (2,84) = 12.68, p <0.01; fear: F (2,84) = 16.24, p <0.01, US expectancy: F (2,84) = 38.28, p<0.01). There was also a significant main effect of CS for valence, arousal, fear, and US expectancy (valence: F (1, 42) = 73.43, p <0.01; arousal: F (1, 42) = 139.40, p <0.01; fear: F (1, 42) = 136.64, p <0.01, US expectancy: F (1, 42) = 169.36, p<0.01). For US expectancy, the CS x tDCS (F (1, 42) = 4.55, p = 0.04) and Phases x CS (F (2, 84) = 6.96, p<0.01) interactions were significant. The respective post-hoc tests showed that the overall valence, arousal, fear, and US expectancy scores were highest for acquisition, then extinction, and were lowest for recall (p<0.01). The overall arousal, fear, and US expectancy scores were greater for the CS+ compared to the CS- (p<0.01), and the overall valence scores were greater for the CS- compared to the CS+ (p<0.01). Irrespective of task phase, US expectancy was significantly greater for the CS+ compared to the CS- for both real and sham tDCS groups (p<0.01), and US expectancy was significantly larger for the CS+ in the sham tDCS compared to the real tDCS condition (p=0.04) (Table S6a, S6b).

**Table S6a.** Valence, arousal, fear, and US expectancy ratings for each stimulus in different conditions for each phase.

|  | | Real tDCS (n=21)  Mean SD | | Sham tDCS (n=23)  Mean SD | |
| --- | --- | --- | --- | --- | --- |
| Valence | | | | | |
| Acquisition | CS+ | 2.90 | 1.73 | 3.00 | 1.73 |
|  | CS- | 6.24 | 2.32 | 5.91 | 1.90 |
| Extinction | CS+ | 4.38 | 1.88 | 3.48 | 1.73 |
|  | CS- | 6.14 | 2.20 | 6.57 | 1.67 |
| Recall | CS+ | 4.86 | 1.71 | 4.09 | 1.81 |
|  | CS- | 7.14 | 1.49 | 7.04 | 1.26 |
| Arousal | | | | | |
| Acquisition | CS+ | 5.33 | 2.08 | 4.96 | 2.42 |
|  | CS- | 1.86 | 2.48 | 1.70 | 2.03 |
| Extinction | CS+ | 5.00 | 1.84 | 5.52 | 1.20 |
|  | CS- | 1.33 | 1.56 | 1.35 | 1.72 |
| Recall | CS+ | 3.48 | 1.86 | 4.48 | 1.70 |
|  | CS- | 0.90 | 1.61 | 0.61 | 1.27 |
| Fear | | | | | |
| Acquisition | CS+ | 4.29 | 2.63 | 5.30 | 1.96 |
|  | CS- | 1.10 | 1.73 | 1.17 | 1.75 |
| Extinction | CS+ | 3.67 | 2.22 | 5.00 | 2.07 |
|  | CS- | 1.19 | 1.91 | 1.04 | 1.55 |
| Recall | CS+ | 2.76 | 2.00 | 3.74 | 2.22 |
|  | CS- | 0.33 | 0.73 | 0.43 | 0.90 |
| US Expectancy | | | | | |
| Acquisition | CS+ | 5.24 | 1.97 | 5.83 | 1.30 |
|  | CS- | 1.76 | 2.30 | 1.22 | 1.70 |
| Extinction | CS+ | 3.71 | 2.26 | 4.70 | 1.99 |
|  | CS- | 1.19 | 1.50 | 1.04 | 1.66 |
| Recall | CS+ | 2.33 | 1.88 | 3.04 | 1.89 |
|  | CS- | 0.52 | 0.98 | 0.43 | 0.84 |

**Table S6b.** Results of the mixed model ANOVAs conducted for valence, arousal, fear, and US expectancy ratings for each stimulus in different conditions for each phase.

|  | F | df, error | p | ŋ² |
| --- | --- | --- | --- | --- |
| Valence | | | | |
| Phases | 23.58 | 2, 84 | **p <0.01** | 0.36 |
| CS | 73.43 | 1, 42 | **p <0.01** | 0.64 |
| tDCS | 0.79 | 1,42 | 0.38 | 0.02 |
| Phases * tDCS | 0.38 | 2, 84 | 0.68 | 0.01 |
| CS * tDCS | 0.68 | 1, 42 | 0.41 | 0.02 |
| Phases * CS | 1.56 | 2, 84 | 0.22 | 0.04 |
| Phases * CS * tDCS | 2.34 | 2, 84 | 0.10 | 0.05 |
| Arousal | | | | |
| Phases | 12.68 | 2, 84 | **p <0.01** | 0.23 |
| CS | 139.40 | 1, 42 | **p <0.01** | 0.77 |
| tDCS | 0.12 | 1,42 | 0.72 | 0.01 |
| Phases * tDCS | 1.03 | 2, 84 | 0.36 | 0.02 |
| CS * tDCS | 0.80 | 1, 42 | 0.38 | 0.02 |
| Phases * CS | 1.17 | 2, 84 | 0.32 | 0.03 |
| Phases * CS * tDCS | 1.23 | 2, 84 | 0.30 | 0.03 |
| Fear | | | | |
| Phases | 16.24 | 2, 84 | **p <0.01** | 0.28 |
| CS | 136.64 | 1, 42 | **p <0.01** | 0.76 |
| tDCS | 2.68 | 1,42 | 0.11 | 0.06 |
| Phases * tDCS | 0.01 | 2, 84 | 0.99 | 0.00 |
| CS * tDCS | 3.91 | 1, 42 | 0.05 | 0.09 |
| Phases * CS | 1.61 | 2, 84 | 0.21 | 0.04 |
| Phases * CS * tDCS | 0.28 | 2, 84 | 0.76 | 0.01 |
| US expectancy | | | | |
| Phases | 34.28 | 2, 84 | **p <0.01** | 0.45 |
| CS | 169.36 | 1, 42 | **p <0.01** | 0.80 |
| tDCS | 0.86 | 1,42 | 0.36 | 0.02 |
| Phases * tDCS | 0.38 | 2, 84 | 0.68 | 0.01 |
| CS * tDCS | 4.55 | 1, 42 | **0.04** | 0.10 |
| Phases * CS | 6.96 | 2, 84 | **p <0.01** | 0.14 |
| Phases * CS * tDCS | 0.08 | 2, 84 | 0.93 | 0.00 |

df: degrees of freedom

**Table S7**: The average magnitude of the simulated electric field (V/m) using the employed tDCS montage in different regions in the real tDCS group using the individual anatomical images. The electric field simulation was done using SimNIBS 4.5 [[3](#_ENREF_3)]

| ROI | vmPFC | Right DLPFC | Left DLPFC | Right Amygdala | Left Amygdala |
| --- | --- | --- | --- | --- | --- |
| average magnitude of the electric field (V/m) | 0.233 | 0.084 | 0.083 | 0.061 | 0.041 |


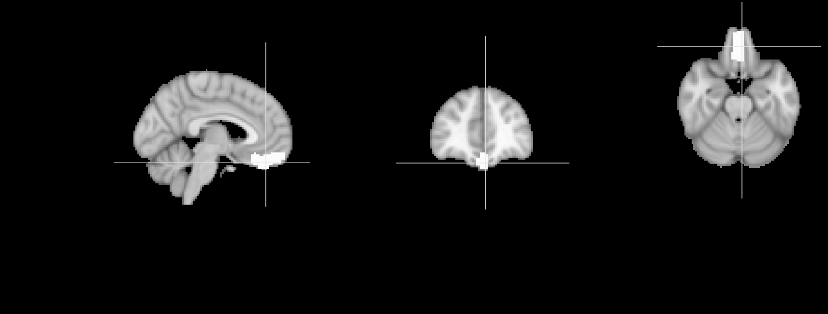


**Fig S3**. vmPFC region of interest (ROI) mask


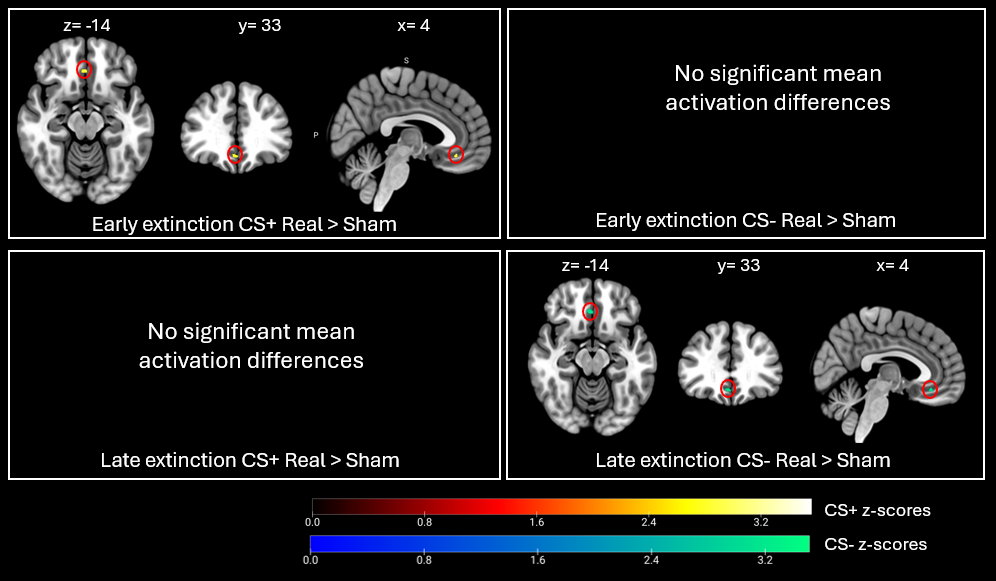


**Fig S4**. CS+ and CS- only activations during extinction for the real > sham tDCS differences of the vmPFC. The clusters (thresholded z-maps) are reported for the early (top row) and late (bottom row) blocks of the experimental phases. All significant clusters are displayed along with the x, y, z coordinates of the slices. Results are displayed on the MNI152 brain. The color bar indicates z-scores, with warm colors (red to white) representing CS+ z-values, and cool colors (blue to green) representing CS- z-values, ranging from 0 to 3.2, where higher values are associated with larger differences.


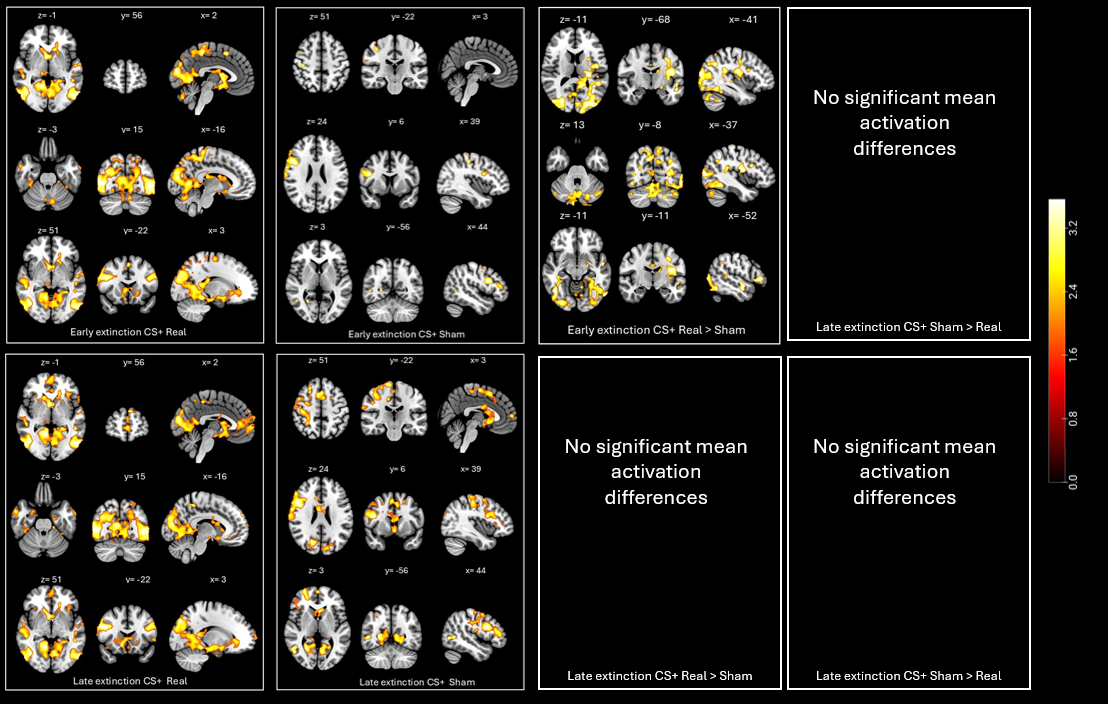


**Fig S5**. CS+ only activations during extinction for the real (first column), and sham (second column) stimulation groups, and real > sham (third column), and sham > real (fourth column) differences. The clusters (thresholded z-maps) are reported for the early (top row) and late (bottom row) blocks of the experimental phases. All significant clusters are displayed along with the x, y, z coordinates of the slices. Results are displayed on the MNI152 brain. The color bar indicates the z-scores ranging from 0 to 3.2, where higher values are associated with larger differences.


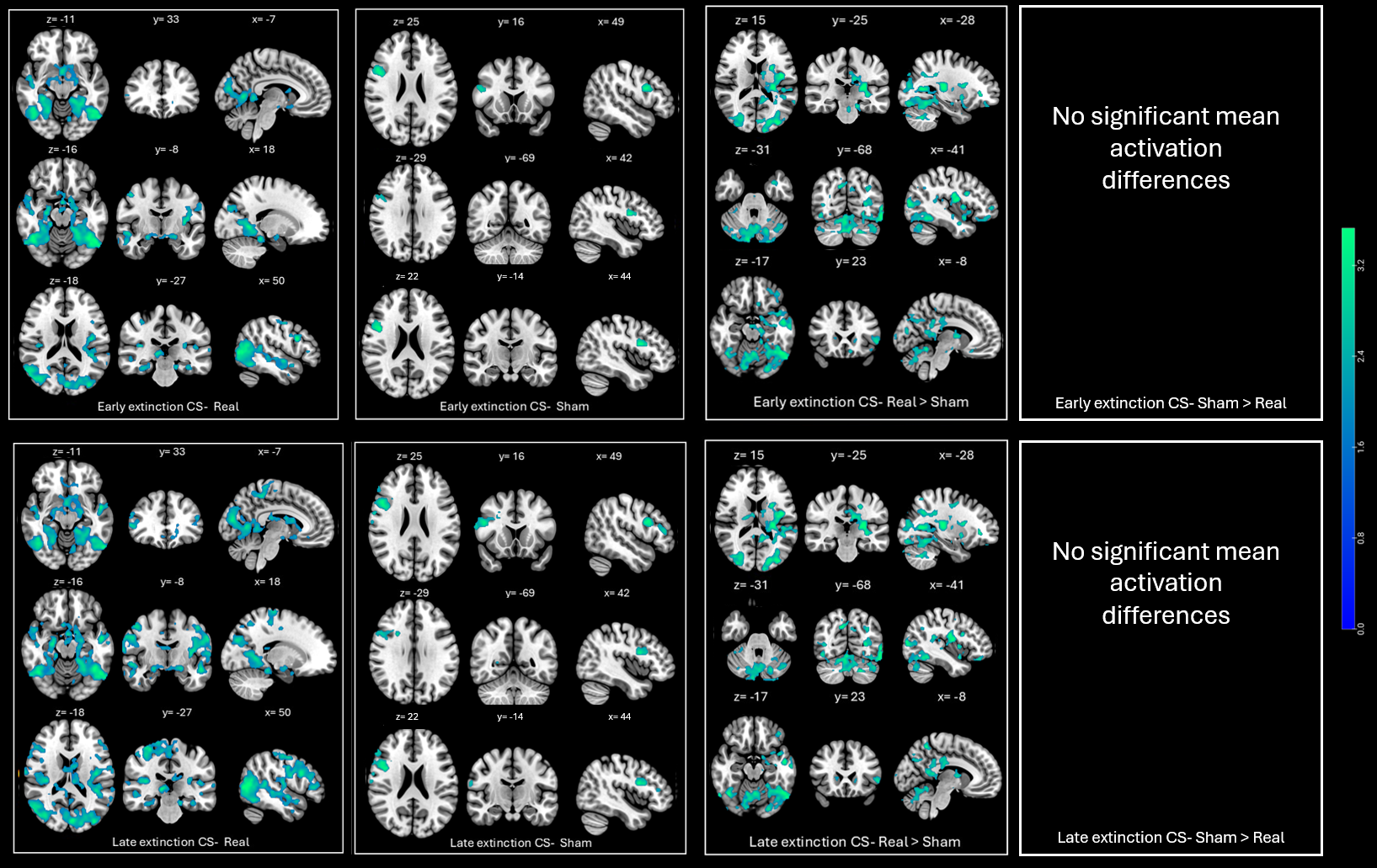


**Fig S6.** CS- only activations during extinction for the real (first column), and sham (second column) stimulation groups, and real > sham (third column), and sham > real (fourth column) differences. The clusters (thresholded z-maps) are reported for the early (top row) and late (bottom row) blocks of the experimental phases. All significant clusters are displayed along with the x, y, z coordinates of the slices. Results are displayed on the MNI152 brain. The color bar indicates the z-scores ranging from 0 to 3.2, where higher values are associated with larger differences.

**Table S8**. Activations for the conditions CS+ and CS- only during extinction. The clusters are reported for the early and late blocks of extinction. All significant clusters are displayed with the local maxima within the cluster. The regions which belong to these local maxima are mentioned but usually these clusters also include other regions beyond the local maxima which are reported as additional regions in the clusters.

| **Voxel cluster (TFCE: Threshold Free Cluster Enhancement)** | **P (FWE)**  **Family-wise error corrected p value** | **z-score** | **global maxima coordinates (mm)** | | | **local maxima within the voxel cluster**  **(L: Left, R: Right, and Bi: Bilateral hemispheres)** |
| --- | --- | --- | --- | --- | --- | --- |
| **x y z** | | | | | | |
| **Early Extinction: CS+ Real tDCS** | | | | | | |
| 35310 | <0.001 | 3.54 | -36 | -48 | -22 | Bi. Lateral Occipital Cortex, R. Inferior Temporal Gyrus, L. Inferior Frontal Gyrus |
| 92 | 0.04 | 3.54 | -2 | 14 | 54 | L. Superior Frontal Gyrus, L. Supplementary Motor Cortex, L. Paracingulate Cortex |
| 19 | 0.05 | 3.16 | 10 | 12 | 46 | L. Paracingulate Cortex, L. Anterior Cingulate Cortex |
| Additional regions in the clusters: Bi. Amygdala, L. Parahippocampal Gyrus, L. Middle Temporal Gyrus, L. Posterior Cingulate Cyrus, L. Insular Cortex, R. Middle Frontal Gyrus, L. Inferior Frontal Gyrus | | | | | | |
| **Early Extinction: CS+ Sham tDCS** | | | | | | |
| 1183 | 0.01 | 3.54 | 44 | 4 | 22 | R. Precentral Gyrus, R. Postcentral Gyrus, R. Inferior Frontal Gyrus |
| 72 | 0.03 | 3.35 | 40 | -24 | 50 | R. Precentral Gyrus, R. Postcentral Gyrus |
| 52 | 0.03 | 3.54 | 34 | -26 | 64 | R. Precentral Gyrus, R. Postcentral Gyrus |
| 40 | 0.05 | 2.79 | 50 | -2 | 44 | R. Precentral Gyrus, R. Middle Frontal Gyrus |
| 33 | 0.03 | 3.54 | 24 | -60 | 2 | R. Lingual Gyrus |
| 16 | 0.05 | 3.35 | 48 | -56 | 2 | R. Middle Temporal Gyrus |
| **Early Extinction: CS+ Real > CS+ Sham tDCS** | | | | | | |
| 12383 | 0.01 | 3.54 | -56 | -66 | -18 | R. Precuneus Cortex, L. Lateral Occipital Cortex |
| 4365 | 0.02 | 3.54 | -24 | -70 | -40 | R. Lateral Occipital Cortex, R. Occipital Pole, R. Occipital Fusiform Gyrus, L. Cerebellum (V) |
| 258 | 0.04 | 3.35 | 2 | 4 | 2 | L. Thalamus, L. Caudate |
| 210 | 0.05 | 3.09 | -46 | -68 | -32 | L. Cerebellum (Crus I) |
| 177 | 0.04 | 3.54 | -54 | 18 | 0 | L. Inferior Frontal Gyrus |
| 151 | 0.04 | 3.54 | -50 | -8 | -16 | L. Superior Temporal Gyrus, L. Middle Temporal Gyrus |
| 43 | 0.05 | 3.54 | 26 | 16 | 0 | R. Putamen, R. Caudate |
| 36 | 0.05 | 3.35 | 24 | -60 | -40 | R. Cerebellum (VI, Crus I) |
| 19 | 0.05 | 2.71 | -2 | -78 | -10 | L. Lingual Gyrus |
| 16 | 0.05 | 3.54 | -22 | -8 | -12 | L. Amygdala |
| 13 | 0.05 | 3.54 | -18 | 0 | -14 | L. Amygdala |
| 6 | 0.05 | 3.24 | 14 | -50 | -40 | R. Cerebellum (IX) |
| Additional regions in the clusters: L. Parahippocampal Gyrus, Bi. Middle Frontal Gyrus, L. Posterior and Anterior Cingulate Cortex, L. Insular Cortex, L. Inferior Frontal Gyrus | | | | | | |
| **Early Extinction: CS+ Sham > CS+ Real tDCS** | | | | | | |
| no significant mean activation differences | | | | | | |
|  | | | | | | |
| **Early Extinction: CS- Real tDCS** | | | | | | |
| 17417 | <0.001 | 3.54 | -36 | -46 | -20 | L. Temporal Occipital Fusiform Cortex, L. Supramarginal Gyrus, L. Lateral Occipital Cortex |
| 403 | 0.02 | 3.54 | 50 | 12 | 24 | R. Precentral Cortex, R. Inferior Frontal Gyrus, R. Middle Frontal Gyrus |
| 378 | 0.03 | 3.35 | 40 | 0 | 40 | R. Precentral Cortex, R. Postcentral Cortex |
| 64 | 0.04 | 3.24 | 38 | -24 | 14 | R. Heschl’s Gyrus, R. Parietal Opercular Cortex |
| 13 | 0.05 | 2.75 | 56 | 32 | 6 | R. Inferior Frontal Gyrus |
| Additional regions in the clusters: Bi. Middle Temporal Gyrus, Bi. Posterior Cingulate Gyrus, Bi. Precuneus, Bi. Amygdala, R. Parahippocampal Gyrus, R. Thalamus, R. Caudate, Bi. Cerebellum | | | | | | |
| **Early Extinction: CS- Sham tDCS** | | | | | | |
| 290 | 0.01 | 3.54 | 44 | 4 | 22 | R. Precentral Gyrus, R. Inferior Frontal Gyrus |
| **Early Extinction: CS- Real > CS- Sham tDCS** | | | | | | |
| 17337 | 0.02 | 3.54 | -28 | -64 | -40 | L. Temporal Occipital Fusiform Cortex, L. Supramarginal Gyrus, L. Lateral Occipital Cortex |
| 204 | 0.05 | 3.54 | -38 | 48 | 0 | L. Subcallosal Cortex, L. Orbitofrontal Cortex, L. Frontal Pole |
| 71 | 0.04 | 3.54 | 26 | 16 | 0 | R. Putamen |
| 26 | 0.05 | 3.16 | 2 | 30 | -10 | R. Subcallosal Cortex |
| 22 | 0.05 | 3.24 | 18 | 2 | -6 | R. Putamen, R. Pallidum |
| 19 | 0.05 | 3.24 | 6 | -64 | 8 | L. Intracalcarine Cortex |
| Additional regions in the clusters: Bi. Middle Temporal Gyrus, Bi. Posterior Cingulate Gyrus, Bi. Amygdala, R. Parahippocampal Gyrus, R. Thalamus, R. Caudate, Bi. Cerebellum, L. Putamen, L. Thalamus | | | | | | |
| **Early Extinction: CS- Sham > CS- Real tDCS** | | | | | | |
| no significant mean activation differences | | | | | | |
|  | | | | | | |
| **Late Extinction: CS+ Real tDCS** | | | | | | |
| 33567 | <0.001 | 3.54 | -50 | -62 | -18 | R. Nucleus Accumbens, Bi. Inferior Frontal Gyrus, L. Lateral Occipital Cortex, R. Cuneal Cortex |
| 663 | 0.03 | 3.24 | 34 | -54 | 50 | R. Superior Parietal Lobule, Bi. Precuneus, L. Postcentral Gyrus, Bi. Posterior Cingulate Cortex, R. Lateral Occipital Cortex, L. Precentral Gyrus |
| 56 | 0.04 | 3.35 | -8 | 60 | 30 | L. Frontal Pole, L. Medial Frontal Cortex |
| 1 | 0.05 | 3.04 | 10 | 12 | 46 | R. Precentral Gyrus |
| 1 | 0.05 | 3.54 | 18 | -22 | 76 | R. Paracingulate Gyrus |
| **Late Extinction: CS+ Sham tDCS** | | | | | | |
| 9679 | 0.02 | 3.54 | 28 | 32 | -18 | R. Inferior Frontal Gyrus, R. Middle Frontal Gyrus, R. Inferior Frontal Gyrus, R. Frontal Pole, R. Medial Frontal Cortex |
| 2117 | 0.01 | 3.54 | 18 | -42 | -6 | R. Lingual Gyrus, R. Cuneal Cortex, R. Posterior Cingulate Cortex |
| 1205 | 0.02 | 3.54 | -8 | -66 | 4 | L. Lingual Gyrus, L. Parahippocampal Gyrus (posterior division), |
| 152 | 0.03 | 3.54 | 48 | -68 | 0 | R. Middle Temporal Gyrus, R. Occipital Cortex |
| 106 | 0.03 | 3.35 | 2 | 60 | 6 | L. Paracingulate Cortex, L. Frontal Pole |
| 7 | 0.05 | 2.48 | 12 | 54 | 18 | R. Superior Frontal Gyrus |
| **Late Extinction: CS+ Real > CS+ Sham tDCS** | | | | | | |
| no significant mean activation differences | | | | | | |
| **Late Extinction: CS+ Sham > CS+ Real tDCS** | | | | | | |
| no significant mean activation differences | | | | | | |
|  | | | | | | |
| **Late Extinction: CS- Real tDCS** | | | | | | |
| 31385 | <0.001 | 3.54 | -50 | -62 | -16 | L. Superior Temporal Gyrus, L. Middle Temporal Gyrus, Bi. Intracalcarine Cortex, L. Supramarginal Gyrus, R. Precentral Gyrus, R. Postcentral Gyrus, L. Cuneal Cortex, L. Lateral Occipital Cortes, L. Inferior Temporal Gyrus |
| **Late Extinction: CS- Sham tDCS** | | | | | | |
| 725 | 0.01 | 3.54 | 44 | 6 | 22 | R. Precentral Gyrus, R. Inferior Frontal Gyrus |
| 38 | 0.04 | 2.73 | 64 | -14 | 24 | R. Postcentral Gyrus |
| 27 | 0.04 | 2.99 | 62 | -2 | 24 | R. Postcentral Gyrus |
| 14 | 0.05 | 3.54 | 26 | -58 | 4 | R. Precentral Gyrus, R. Postcentral Gyrus |
| 13 | 0.04 | 3.35 | 34 | -28 | 66 | R. Precentral Gyrus, R. Postcentral Gyrus |
| 7 | 0.05 | 3.09 | 22 | 36 | 34 | R. Frontal Pole |
| **Late Extinction: CS- Real > CS- Sham tDCS** | | | | | | |
| 13937 | 0.02 | 3.54 | -22 | -68 | -40 | L. Cerebellum (V), L. Inferior Temporal Gyrus, L. Lateral Occipital Cortex, L. Lateral Occipital Fusiform Gyrus, R. Cerebellum (V,VI) |
| 697 | 0.04 | 3.54 | -20 | 16 | 0 | L. Central Opercular Cortex, L. Insular Cortex, L. Inferior Frontal Gyrus, L. Putamen |
| 524 | 0.04 | 3.54 | 14 | -70 | 36 | L. Lateral Occipital Cortex, Bi. Precuneus Cortex |
| 517 | 0.03 | 3.54 | -62 | -2 | -26 | L. Superior Temporal Gyrus, L. Middle Temporal Gyrus, L. Heschl's Gyrus |
| 246 | 0.04 | 3.54 | -8 | -54 | 56 | L. Lateral Occipital Cortex, L. Precuneus Cortex |
| 211 | 0.03 | 3.54 | -22 | -10 | -14 | L. Amygdala |
| 131 | 0.04 | 3.35 | -42 | 4 | -16 | L. Orbitofrontal Cortex, L. Insular Cortex, L. Temporal Pole |
| 104 | 0.04 | 3.24 | 16 | -58 | 54 | R. Superior Parietal Lobule, R. Precuneus Cortex, R. Lateral Occipital Cortex |
| 81 | 0.04 | 3.54 | 26 | 16 | 0 | R. Caudate, R. Putamen |
| 78 | 0.04 | 3.54 | 58 | -62 | -20 | R. Inferior Temporal Gyrus |
| 68 | 0.05 | 3.09 | -38 | 36 | -18 | L. Frontal Pole, L. Orbitofrontal Cortex |
| 55 | 0.04 | 3.35 | 12 | -50 | 30 | R. Posterior Cingulate Gyrus |
| 30 | 0.04 | 3.35 | 20 | 4 | -6 | R. Putamen, R. Pallidum |
| 12 | 0.05 | 3.09 | 12 | -40 | 48 | R. Precuneus Cortex |
| 10 | 0.05 | 3.24 | 60 | -62 | 4 | R. Lateral Occipital Cortex, R. Middle Temporal Gyrus |
| 7 | 0.05 | 2.79 | -26 | 48 | -20 | L. Frontal Pole |
| 7 | 0.05 | 3.54 | -12 | -66 | 56 | L. Lateral Occipital Cortex |
| 7 | 0.05 | 3.54 | -18 | -30 | -26 | L. Cerebellum (I-VI) |
| 3 | 0.05 | 2.99 | -2 | -34 | 54 | L. Precentral Gyrus |
| 3 | 0.05 | 3.16 | -14 | -22 | -18 | Brainstem |
| **Late Extinction: CS- Sham > CS- Real tDCS** | | | | | | |
| no significant mean activation differences | | | | | | |


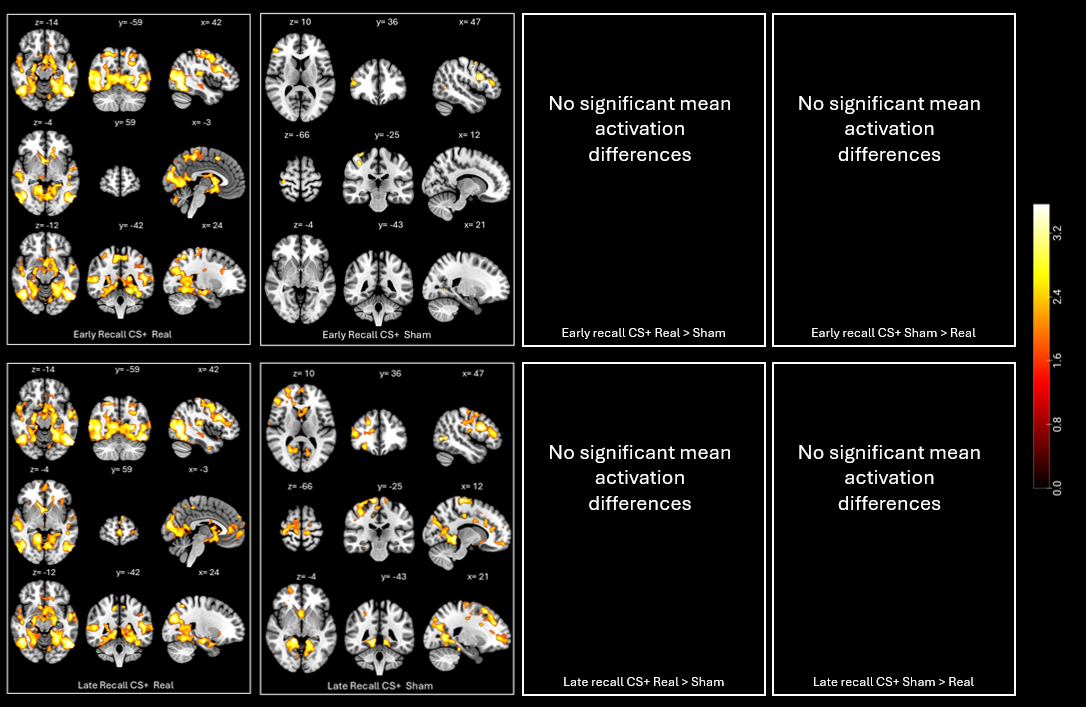


**FigS7.** CS+ only activations during recall for the real (first column), and sham (second column) stimulation groups, and real > sham (third column), and sham > real (fourth column) differences. The clusters (thresholded z-maps) are reported for the early (top row) and late (bottom row) blocks of the experimental phases. All significant clusters are displayed along with the x, y, z coordinates of the slices. Results are displayed on the MNI152 brain. The color bar indicates the z-scores ranging from 0 to 3.2, where higher values are associated with larger differences.


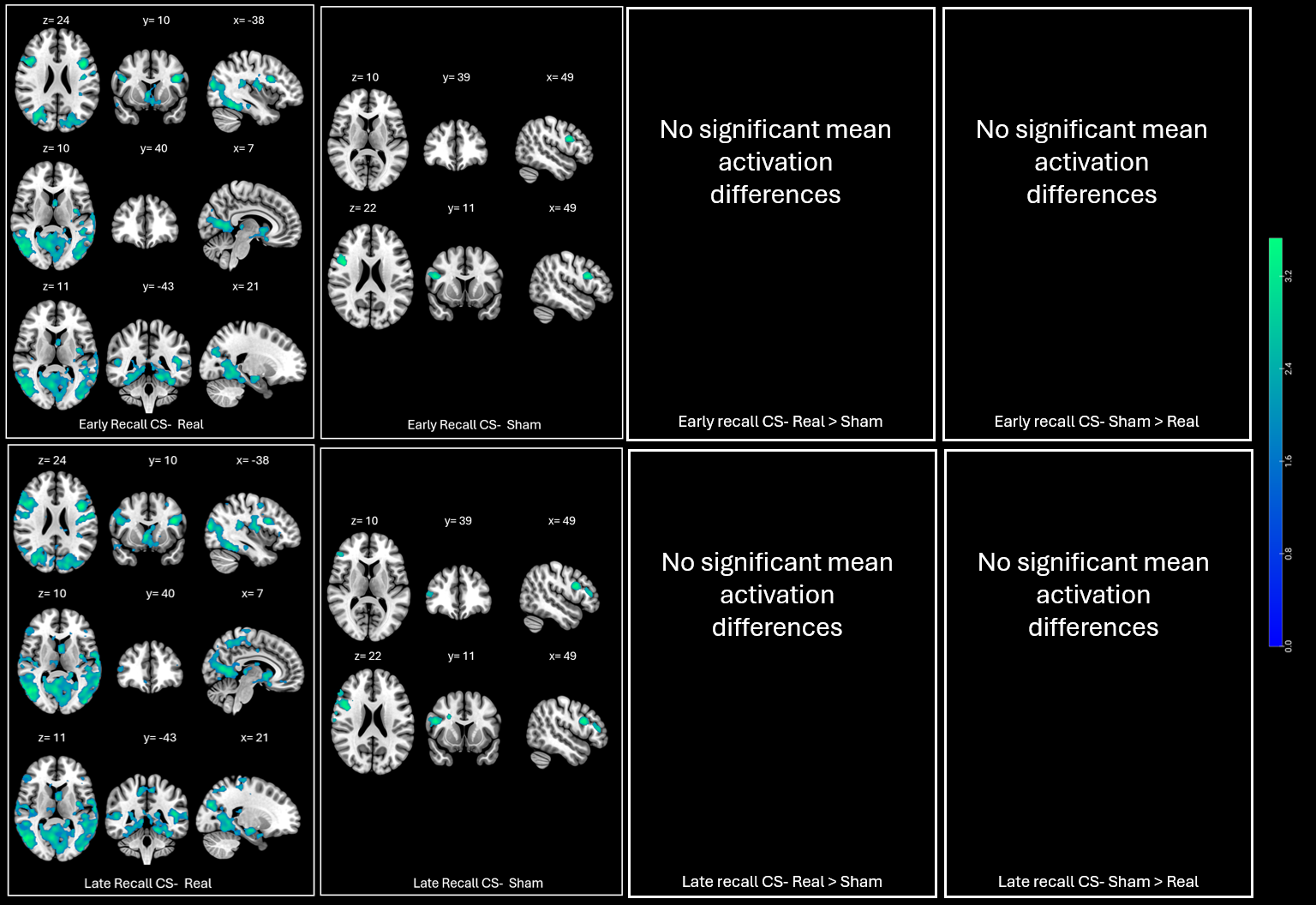


**FigS8.** CS- only activations during recall for the real (first column), and sham (second column) stimulation groups, and real > sham (third column), and sham > real (fourth column) differences. The clusters (thresholded z-maps) are reported for the early (top row) and late (bottom row) blocks of the experimental phases. All significant clusters are displayed along with the x, y, z coordinates of the slices. Results are displayed on the MNI152 brain. The color bar indicates the z-scores ranging from 0 to 3.2, where higher values are associated with larger differences.

**Table S9.** Activations for the conditions CS+ and CS- only during recall. The clusters are reported for the early and late blocks of extinction. All significant clusters are displayed with the local maxima within the cluster. The regions which belong to these local maxima are mentioned but usually these clusters also include other regions beyond the local maxima which are reported as additional regions in the clusters.

| **Voxel cluster (TFCE: Threshold Free Cluster Enhancement)** | **P (FWE)**  **Family-wise error corrected p value** | **z-score** | **global maxima coordinates (mm)** | | | **local maxima within the voxel cluster**  **(L: Left, R: Right, and Bi: Bilateral hemispheres)** |
| --- | --- | --- | --- | --- | --- | --- |
| **x y z** | | | | | | |
| **Early Recall: CS+ Real tDCS** | | | | | | |
| 9264 | <0.001 | 3.54 | 44 | -48 | -20 | Bi. Lateral Occipital Cortex, R. Middle Temporal Gyrus |
| 834 | <0.001 | 3.54 | 30 | -56 | 42 | R. Supramarginal Gyrus, R. Superior Parietal Lobule, R. Postcentral Gyrus, R. Angular Gyrus |
| 15 | 0.05 | 2.62 | 20 | -70 | 50 | R. Lateral Occipital Cortex |
| 13 | 0.05 | 2.54 | -38 | -44 | -26 | L. Temporal Fusiform Cortex, L. Cerebellum (VI) |
| 6 | 0.05 | 2.59 | 10 | -86 | 16 | R. Cuneal Cortex |
| **Early Recall: CS+ Sham tDCS** | | | | | | |
| 15860 | <0.001 | 3.54 | 22 | -50 | -10 | Bi. Lateral Occipital Cortex, R. Lingual Gyrus, R. Thalamus, R. Hippocampus, R. Posterior Cingulate Gyrus |
| 139 | 0.03 | 3.24 | 58 | 12 | 26 | R. Precentral Gyrus |
| **Early Recall: CS+ Real > CS+ Sham tDCS** | | | | | | |
| no significant mean activation differences | | | | | | |
| **Early Recall: CS+ Sham > CS+ Real** | | | | | | |
| no significant mean activation differences | | | | | | |
|  | | | | | | |
| **Early Recall: CS- Real tDCS** | | | | | | |
| 13790 | <0.001 | 3.54 | 36 | -38 | -24 | Bi. Lateral Occipital Cortex, R. Temporal Fusiform Cortex, R. Superior Parietal Lobule, R. Angular Gyrus |
| 48 | 0.04 | 2.99 | -34 | -42 | -26 | L. Temporal Fusiform Cortex, L. Cerebellum (V, VI) |
| 36 | 0.04 | 3.16 | 10 | -34 | 34 | R. Posterior Cingulate Gyrus |
| 25 | 0.05 | 2.67 | -40 | -52 | 10 | L. Middle Temporal Gyrus |
| 3 | 0.05 | 2.75 | 24 | -18 | 72 | R. Precentral Gyrus |
| **Early Recall CS- Sham tDCS** significant mean activation | | | | | | |
| 17296 | <0.001 | 3.54 | 28 | -34 | -22 | Bi. Lingual Gyrus, R. Middle Temporal Gyrus, R. Lateral Occipital Cortex, R. Superior Parietal Lobule |
| 15 | 0.05 | 3.35 | 50 | 14 | 24 | R. Precentral Gyrus, R. Inferior Frontal Gyrus |
| **Early Recall: CS- Real > CS- Sham tDCS** | | | | | | |
| no significant mean activation differences | | | | | | |
| **Early Recall: CS- Sham > CS- Real** | | | | | | |
| no significant mean activation differences | | | | | | |
|  | | | | | | |
| **Late Recall: CS+ Real tDCS** | | | | | | |
| 10807 | <0.001 | 3.54 | 46 | -52 | -20 | R. Middle Temporal Gyrus, L. Lingual Gyrus, Bi. Lateral Occipital Cortex |
| 96 | 0.03 | 3.54 | 44 | -14 | 40 | R. Precentral Gyrus, R. Postcentral Gyrus, R. Middle Frontal Gyrus |
| 42 | 0.04 | 3.16 | 44 | 12 | 26 | R. Precentral Gyrus, R. Inferior Frontal Gyrus |
| **Late Recall: CS+ Sham tDCS** | | | | | | |
| 15484 | <0.001 | 3.54 | 24 | -48 | -10 | R. Precentral Gyrus, Bi. Posterior Cingulate Gyrus, Bi. Supplementary Motor, R. Lateral Occipital Cortex, R. Supramarginal Gyrus, R. Middle Temporal Gyrus, R. Lingual Gyrus |
| 88 | 0.04 | 2.62 | 60 | -6 | 32 | R. Precentral Gyrus, R. Postcentral Gyrus, R. Supramarginal Gyrus |
| 39 | 0.04 | 3.35 | 58 | 12 | 26 | R. Precentral Gyrus, R. Inferior Frontal Gyrus |
| 24 | 0.04 | 3.35 | -6 | -12 | 20 | L. Thalamus |
| 4 | 0.04 | 2.73 | -8 | -4 | 42 | L. Anterior Cingulate Gyrus, L. Supplementary Motor |
| **Late Recall: CS+ Real > CS+ Sham tDCS** | | | | | | |
| no significant mean activation differences | | | | | | |
| **Late Recall: CS+ Sham > CS+ Real tDCS** | | | | | | |
| no significant mean activation differences | | | | | | |
|  | | | | | | |
| **Late Recall: CS- Real tDCS** | | | | | | |
| 6281 | <0.001 | 3.54 | 44 | -50 | -20 | R. Temporal Occipital Fusiform Cortex, R. Intracalcarine Cortex, |
| 1815 | 0.01 | 3.54 | -44 | -72 | -2 | L. Lateral Occipital Cortex |
| 535 | 0.02 | 3.35 | 46 | 12 | 26 | R. Precentral Gyrus, R. Postcentral Gyrus, R. Inferior Frontal Gyrus |
| 6 | 0.05 | 2.77 | 38 | -22 | 56 | R. Precentral Gyrus, R. Postcentral Gyrus |
| **Late Recall: CS- Sham tDCS** | | | | | | |
| 14775 | <0.001 | 3.54 | 28 | -34 | -22 | L. Lingual Gyrus, Bi. Lateral Occipital Cortex, R. Thalamus, R. Posterior Cingulate Gyrus |
| 3 | 0.05 | 2.35 | -4 | -56 | 48 | L. Precuneus Cortex |
| **Late Recall: CS- Real > CS- Sham tDCS** | | | | | | |
| no significant mean activation differences | | | | | | |
| **Late Recall: CS- Sham > CS- Real tDCS** | | | | | | |
| no significant mean activation differences | | | | | | |


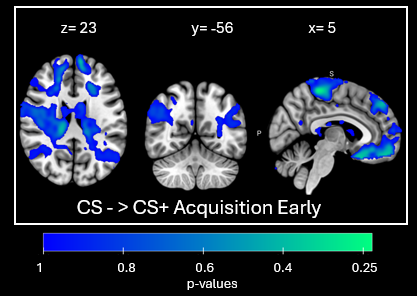


**Fig S9**. CS- versus CS+ activations during fear acquisition. The clusters (p-stats map) are reported for the early block of fear acquisition. All significant clusters are displayed along with the x, y, z coordinates of the slices. Results are displayed in the MNI152 brain. The color bar indicates the p-values, ranging from 1 to 0.25, where lower values are associated with larger differences.

Supplementary PPI results for CS+ and CS- for real < sham tDCS

In the early block, significant functional connectivity was only observed for CS+ for real < sham tDCS. Compared to sham tDCS, real tDCS resulted in a significantly stronger functional decoupling between the vmPFC and the left temporal gyrus, regions within the right and left occipital cortex, right posterior cingulate cortex, right cuneal cortex, and right precuneus. For the late block, significant functional connectivity was observed for both CS+ and CS- for real < sham tDCS. For CS+, compared to sham tDCS, real tDCS significantly resulted in a stronger functional decoupling between the vmPFC and the left supramarginal gyrus, left superior parietal lobule, right superior frontal gyrus, and the bilateral cerebellum. For CS-, compared to sham tDCS, real tDCS significantly reduced functional connectivity between the vmPFC and bilateral lingual gyrus, bilateral intracalcarine cortex, and left occipital pole (Table S10 and Fig. S10).


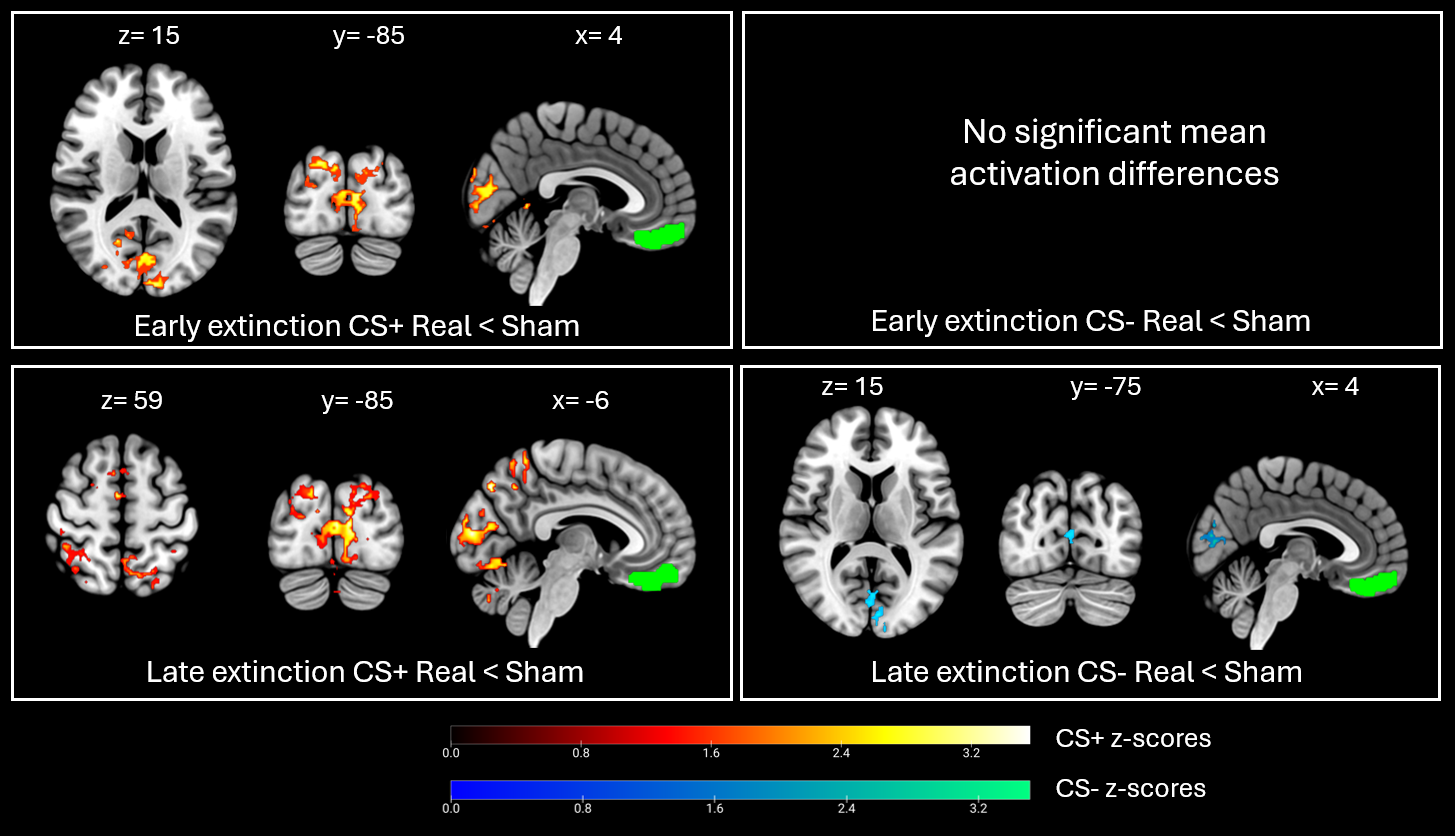


**Fig S10.** CS+ and CS- seed-based functional connectivity analysis during extinction for real versus sham tDCS (z-maps). Bilateral vmPFC was used as the seed for this analysis (seed mask shown in green color). Threshold-free cluster enhancement (TFCE) is shown with a p threshold of 0.05. Anatomical labels were assigned with the Harvard-Oxford cortical and subcortical structural atlas (Harvard-Oxford Cortical Structural Atlas (RRID:SCR_001476)) and Cerebellar Altas in MNI152 space after normalization with FLIRT. Connectivity results are displayed in the MNI152 brain. The color bar indicates z-scores, with warm colors (red to white) representing CS+ z-values, and cool colors (blue to green) representing CS- z-values ranging from 0 to 3.2, where higher values are associated with larger differences.

**Table S10.** CS+ and CS- seed-based functional connectivity analysis for sham versus real tDCS during the extinction phase. The clusters are reported for the early and late blocks. All significant clusters are displayed along with regions in the clusters, local maxima are reported in MNI coordinates. For the larger clusters, additional regions with significant activations beyond local maxima are also reported.

| **Voxel cluster (TFCE: Threshold Free Cluster Enhancement)** | **P (FWE)Family-wise error corrected p value** | **z-score** | **Voxel Cluster Global Maxima Coordinate in MNI (mm)** | | | **Local Maxima within the Voxel Cluster**  **(L: Left, R: Right, and Bi: Bilateral hemispheres)** |
| --- | --- | --- | --- | --- | --- | --- |
| **x y z** | | | | | | |
| **Early Extinction: CS+ Real < CS+ Sham tDCS** | | | | | | |
| 1249 | 0.047 | 3.54 | 8 | -50 | 2 | L. Cuneal Cortex, R. Occipital Pole, R. Lingual Gyrus, R. Posterior Cingulate Cortex, R. Supracalcarine Cortex, R. Intracalcarine Cortex, R. Lateral Occipital Cortex |
| 1 | 0.047 | 2.73 | -46 | -50 | -18 | L. Inferior Temporal Gyrus |
| 1 | 0.049 | 3.54 | -8 | -58 | 2 | L. Lingual Gyrus |
| Additional regions in the clusters: R. Planum Temporale, L. Lateral Occipital Cortex, L. Temporal Occipital Fusiform Cortex, L. Inferior Temporal Gyrus, R. Precuneus | | | | | | |
| **Early Extinction: CS- Real < CS- Sham tDCS** | | | | | | |
| no significant mean activation differences | | | | | | |
| **Late Extinction: CS+ Real < CS+ Sham tDCS** | | | | | | |
| 2965 | 0.029 | 3.54 | -6 | -90 | 10 | L. Precuneus, L. Lateral Occipital Cortex, L. Supracalcarine Cortex, L. Occipital Pole |
| 97 | 0.040 | 3.54 | -36 | -50 | 44 | L. Supramarginal Gyrus (posterior division), L. Superior Parietal Lobule, L. Postcentral Gyrus |
| 42 | 0.048 | 3.35 | -62 | -10 | 16 | L. Planum Temporale, L. Superior Temporal Pole |
| 37 | 0.048 | 3.54 | -56 | -16 | 24 | L. Postcentral Gyrus, L. Central Operculum |
| 18 | 0.045 | 3.54 | 4 | -8 | 60 | L. Supplementary Motor Cortex |
| 17 | 0.049 | 3.04 | -20 | -66 | -24 | L. Occipital Fusiform Gyrus |
| 12 | 0.050 | 3.16 | -12 | -72 | -6 | L. Lingual Gyrus |
| 7 | 0.043 | 2.77 | -56 | -28 | 28 | L. Supramarginal Gyrus (anterior division) |
| Additional regions in the clusters: Bi. Cerebellum, R. Paracingulate Gyrus, R. Superior Frontal Gyrus, L. Precentral Gyrus | | | | | | |
| **Late Extinction: CS- Real < CS- Sham tDCS** | | | | | | |
| 126 | 0.027 | 3.54 | 0 | -72 | 6 | Bi. Lingual Gyrus, Bi. Intracalcarine Cortex |
| 36 | 0.043 | 3.54 | -18 | -96 | 20 | L. Occipital Pole |

**References**

[1] Milad MR, Wright CI, Orr SP, Pitman RK, Quirk GJ, Rauch SL. Recall of fear extinction in humans activates the ventromedial prefrontal cortex and hippocampus in concert. Biol Psychiatry 2007;62(5):446-54.

[2] Oostenveld R, Praamstra P. The five percent electrode system for high-resolution EEG and ERP measurements. Clinical Neurophysiology 2001;112(4):713-9.

[3] Thielscher A, Antunes A, Saturnino GB. Field modeling for transcranial magnetic stimulation: A useful tool to understand the physiological effects of TMS? Annu Int Conf IEEE Eng Med Biol Soc 2015;2015:222-5.

[4] Crawford JR, Henry JD. The positive and negative affect schedule (PANAS): construct validity, measurement properties and normative data in a large non-clinical sample. Br J Clin Psychol 2004;43(Pt 3):245-65.

[5] Brunoni AR, Amadera J, Berbel B, Volz MS, Rizzerio BG, Fregni F. A systematic review on reporting and assessment of adverse effects associated with transcranial direct current stimulation. Int J Neuropsychopharmacol 2011;14(8):1133-45.
